# Shaking Things from the Ground-Up: A Systematic Overview of the Mechanochemistry of Hard and High-Melting Inorganic Materials

**DOI:** 10.3390/molecules28020897

**Published:** 2023-01-16

**Authors:** Thomas Auvray, Tomislav Friščić

**Affiliations:** School of Chemistry, University of Birmingham, Edgbaston, Birmingham B15 2TT, UK

**Keywords:** mechanochemistry, inorganic materials, hydrides, oxides, sulphides, nitrides, borides, green chemistry, solvent-free chemistry, energy materials

## Abstract

We provide a systematic overview of the mechanochemical reactions of inorganic solids, notably simple binary compounds, such as oxides, nitrides, carbides, sulphides, phosphides, hydrides, borides, borane derivatives, and related systems. Whereas the solid state has been traditionally considered to be of little synthetic value by the broader community of synthetic chemists, the solid-state community, and in particular researchers focusing on the reactions of inorganic materials, have thrived in building a rich and dynamic research field based on mechanically-driven transformations of inorganic substances typically seen as inert and high-melting. This review provides an insight into the chemical richness of such mechanochemical reactions and, at the same time, offers their tentative categorisation based on transformation type, resulting in seven distinct groupings: (*i*) the formation of adducts, (*ii*) the reactions of dehydration; (*iii*) oxidation–reduction (redox) reactions; (*iv*) metathesis (or exchange) reactions; (*v*) doping and structural rearrangements, including reactions involving the reaction vessel (the milling jar); (*vi*) acid–base reactions, and (*vii*) other, mixed type reactions. At the same time, we offer a parallel description of inorganic mechanochemical reactions depending on the reaction conditions, as those that: (*i*) take place under mild conditions (e.g., manual grinding using a mortar and a pestle); (*ii*) proceed gradually under mechanical milling; (*iii*) are self-sustained and initiated by mechanical milling, i.e., mechanically induced self-propagating reactions (MSRs); and (*iv*) proceed only via harsh grinding and are a result of chemical reactivity under strongly non-equilibrium conditions. By elaborating on typical examples and general principles in the mechanochemistry of hard and high-melting substances, this review provides a suitable complement to the existing literature, focusing on the properties and mechanochemical reactions of inorganic solids, such as nanomaterials and catalysts.

## 1. Introduction

The mechanochemical transformations of inorganic compounds are of historical significance, as they represent some of the oldest applications of mechanochemical (or tribochemical) phenomena in chemical synthesis and materials processing. Indeed, Takacs proposes that the mechanochemical transformation of mercury(II) sulphide (cinnabar, HgS) into elementary mercury by rubbing the mineral with vinegar in a bronze or copper vessel, described by Theophrastus in the 4th century B.C., represents the oldest known written description of a chemical extraction of a metal from its ore [1,2]. The historical significance of the reactivity of inorganic substances for the development of mechanochemistry is contained in the early mechanochemical studies of Faraday and of Carey Lea [3]. The latter is credited with being the first to clearly demonstrate a qualitative difference between the chemical consequences of a mechanical force acting on a substance, and the results of thermo- or photo-chemical treatment. Over the past few decades, mechanochemistry has rapidly developed into a highly promising methodology for conducting new, cleaner, and generally more applicable solventless transformations [4,5,6,7,8]. Such evolution of mechanochemistry from a laboratory curiosity to a broadly applicable tool of synthesis has led to exciting developments in the areas of crystal engineering [9,10,11], organic [12,13,14,15,16] and bio-organic synthesis [17,18,19], polymer chemistry [20], inorganic chemistry (including traditional inorganic materials, molecular compounds of the main group elements, as well as coordination complexes and polymers) [21,22,23,24,25,26,27,28], as well as catalysis [29,30,31], including enzymatic transformations [32]. An important benefit of mechanochemically-assisted transformations is the opportunity to access previously inaccessible chemical spaces, sometimes through reaction pathways that differ from those observed in classical thermally-activated reactions. The numerous synthetic opportunities offered by mechanochemistry have been documented extensively in the context of organic synthesis by mechanochemical methods [33,34,35,36,37,38,39,40], and the underlying mechanisms are being unravelled through emergent approaches for real-time monitoring of mechanochemical transformations [41,42].

The mechanochemical transformations of substances traditionally perceived as inorganic (e.g., metal oxides, nitrides etc.) are notably distinguished from other areas of mechanosynthesis by involving hard and high-melting solids held together by strong ionic forces and/or extended covalent networks. Within the rich landscape of mechanochemically accessible transformations, the mechanochemistry of inorganic substances stands out as an area heavily populated with materials whose melting points range between ca. 800 °C and 3000 °C and are stabilized by lattice energies of 2–20 MJ mol^−1^, i.e., up to two orders of magnitude larger than transformations of covalent bonds dominant in organic mechanochemistry. Thus, from a very simplified point of view, inorganic mechanochemistry can be regarded as the *mechanochemistry of hard and high-melting materials*.

A number of books and review articles have previously addressed inorganic milling reactions in the contexts of potential mechanisms [43,44], synthetic chemistry with emphasis on nanomaterials [45,46,47,48], as well as alloying and mineral processing [49,50]. There appears, however, to be a gap regarding summarising and a general understanding of the transformations of simple, binary, and often refractory inorganic compounds. As that is a rich area with a long tradition, it would be difficult to provide a detailed and all-encompassing overview. Consequently, instead of mesmerizing the reader with a too wide variety of inorganic mechanochemical reactions, this review is aimed towards highlighting generalizations and mechanistic insights made available through modern, multi-faceted research approaches. We will also attempt to entice the synthetic inorganic chemist or materials scientist by focusing on examples of mechanochemical reactions that contrast with conventional chemist’s experience, illustrating routes to materials and compounds whose preparation by conventional means is difficult or perhaps even impossible, and that also allow us to create conceptual links to other areas of mechanochemistry. For example, although inorganic mechanochemical transformations are dominated by the chemistry of infinite solids, such as metals, metal oxides, and chalcogenides, it is important to remember that molecular substances, such as nonmetal oxides (e.g., phosphorus pentoxide, P_4_O_10_ or carbon dioxide, CO_2_) and non-metals (e.g., elemental sulphur or nitrogen, S_8_ or N_2_) in general are also known to participate, providing at least a conceptual link to mechanochemical reactions yielding metal–organic materials from combinations of inorganic and molecular organic components.

Our overview of inorganic mechanochemical reactions is organized by seven types of transformations:

(1) *adduct formation*: particularly reactions in which there is no formal change in the oxidation number of the reactants.
(2) *dehydration reactions*: mostly transformations of hydrated metal oxides and hydroxides into metal oxides, taking place under mechanical treatment at nominal room temperature.
(3) *reduction-oxidation (redox) reactions*: such as the oxidation of copper metal into copper(II) sulphide by milling with elemental sulphur.
(4) *exchange (metathesis) reactions*: for example, certain reactions observed upon pressing of KBr tablets for infrared spectroscopy analysis.
(5) *doping and structural rearrangements*: including reactions with the mechanochemical reaction assembly (milling jar and/or balls).
(6) *acid–base reactions*: the formation of mixed substances (e.g., phosphates, silicates, mixed metal oxides, inorganic frameworks), typically by reactions of acid and basic oxides
(7) *mixed reactions*: for example, the synthesis of open zeolite frameworks through a combination of acid–base and dehydration processes; the formation of complex oxides (including battery materials, such as LiMn_2_O_4_) through combining different types of reactions; or the transformations of inorganic hydrides via the complex combinations of acid–base, metathesis, and redox chemistry, or even polymorphic transformations

Whereas this categorization of reaction types is, admittedly, somewhat artificial, it works surprisingly well considering the immense diversity of inorganic transformations conducted by milling, manual grinding, or rubbing. We also provide a parallel description of inorganic mechanochemical reactions, depending on the reaction conditions, as those that: (i) take place under conditions that are as mild as manual grinding in a mortar and pestle, (ii) those that proceed gradually under the conditions of continuous mechanical milling, (iii) those that are considered self-sustained and are initiated by mechanical milling (mechanochemical self-propagating reactions, MSRs), and (iv) those that proceed only under conditions of harsh grinding and result from chemical reactivity under strongly non-equilibrium conditions. Particular attention is paid to distinguishing whether a mechanochemical reaction involves a liquid phase or a gas. The latter type of reactivity can either involve a gaseous reactant, for example oxygen, hydrogen, nitrogen, carbon monoxide or dioxide, or a gaseous product, which is, in many cases, carbon dioxide. The participation of a gas in the mechanochemical reaction allows its course to be monitored by following the changes in pressure within the reaction volume, as shown in various studies which have recently encompassed organic transformations and the syntheses of metal–organic framework (MOF) materials [51,52,53].

## 2. Reactions of Adduct Formation

**Scheme 1:**
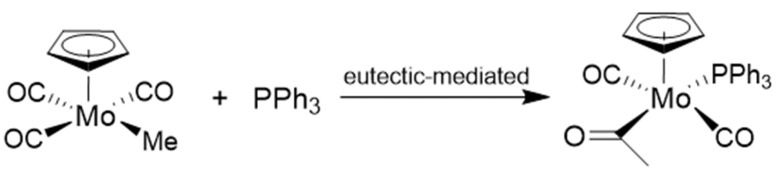
An example of an adduct-forming reaction, reported by Coville and coworkers [55].

Adduct formation is a reaction in which the total mass of reactants is retained within the product. Such reactions occupy an important place in green chemistry, as they represent transformations with ideal atom efficiency, i.e., the reaction product encompasses all reactant atoms used for its manufacture [54]. A number of inorganic mechanochemical reactions can be interpreted as adduct formation, but they are often more conveniently discussed under different topics. For example, the synthesis of complex metal oxides by milling simpler binary (two-component) oxides together or the analogous syntheses of complex metal hydrides can fit into the above definition of adduct formation. However, such reactions should more conveniently be catalogued as acid–base or redox reactions. Another type of reaction that conforms to the definition of an adduct formation is the formation of coordination polymers and discrete molecular complexes through the addition of ligand molecules onto metal precursors, such as metal halides. Such reactivity is best described in the context of coordination chemistry and the preparation of coordination polymers and frameworks. Another type of adduct-forming mechanochemical reaction, in which covalent bonds are formed between neutral molecular reactants, are the syntheses of metal carbonyls by milling the reactive metal in the presence of carbon monoxide, or the addition of neutral two-electron ligands (phosphines, carbon monoxide, alkenes) ligands onto organometallic complexes. In many cases, reactions of the latter type proceed through the formation of a short-lived eutectic intermediate phase. An example of such a reaction is the addition of triphenylphosphine on [(η^5^-Cp)Fe(Me)(CO)_2_] (where Cp is cyclopentadienyl, Me is methyl) or on [(η^5^-Cp)Mo(Me)(CO)_3_], leading to the migratory insertion of a carbonyl ligand (Scheme 1) [55].

## 3. Dehydration Reactions

**Figure 1:**
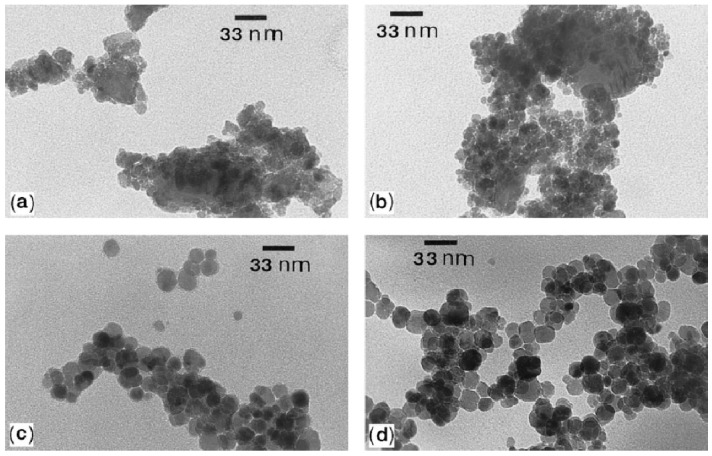
TEM images of a sample of γ-FeOOH during mechanochemical dehydration to hematite after: (**a**) 6 h, (**b**) 18 h, (**c**) 58 h, and (**d**) 70 h milling [57].

Mechanochemical action has a profound effect on inorganic substances lined with hydroxyl (OH) groups, such as clays and hydroxides. Grinding or milling such materials is known to sometimes lead to dehydration, yielding metal oxides. This effect has been extensively studied for the dehydration of iron and other metal hydroxides. An example is the orthorhombic iron(III) oxide hydroxide γ-FeOOH, which upon milling transforms into the hexagonal form of the anhydrous oxide haematite, α-Fe_2_O_3_. According to Klissurski and Blaskov, dehydration by automated grinding in an agate mortar is complete within 60 h. The reaction is particularly interesting because the mechanochemical reaction takes a different course from the thermochemical one [56]. The thermal dehydration of γ-FeOOH yields the oxide in its orthorhombic form γ-Fe_2_O_3_ through a topotactic process, i.e., one in which the crystal structure of the product is geometrically associated to the crystal structure of the reactant. Mechanochemical and thermochemical pathways are thought to be different because mechanical impact and agitation disrupt the topotactic nature of the transformation. Indeed, a more recent investigation of haematite obtained by the mechanical dehydration of α-FeOOH revealed nanosized particles, consistent with the disruption of the crystallization process at the nano (atomic) scale (Figure 1) [57].

Mechanochemical dehydration has also been observed for a variety of other hydroxides, including Cu(OH)_2_ [58], Al(OH)_3_, Zr(OH)_4_ [59] and, if milling is carried out in the presence of silicon dioxide, for Mg(OH)_2_ and Ca(OH)_2_ [60]. The simplest explanation for the mechanochemical dehydration of hydroxide materials is that mechanical impact facilitates the condensation reaction between the nearest-neighbour hydroxylated species, according to Equation (1):

M_1_-OH + HO-M_2_ → M_1_-O-M_2_ + H_2_O (1)

Even the simplistic view of Equation (1) highlights an important synthetic opportunity: that M_1_ and M_2_ do not need to be the same element. Indeed, the mechanochemical treatment of the mixtures of different hydroxides often gives rise to complex (mixed) metal oxides either immediately upon grinding or upon thermal treatment after grinding. Examples of such reactivity include the milling of hydrated TiO_2_ and Mg(OH)_2_ to yield magnesium titanate on subsequent heating, and the milling of Al(OH)_3_ together with hydrated silica to synthesize the important refractory aluminosilicate mullite at a temperature lower than that required for a direct reaction of a mixture of Al_2_O_3_ and SiO_2_ (1200 °C vs. 1400 °C). These observations are all consistent with the incipient formation of Ti-O-Mg or Si-O-Al linkages upon milling, which facilitate the subsequent formation of complex materials. Such considerations led to an important new concept in inorganic mechanochemistry in the early 1990s, when it was recognized by a number of authors that, besides traditional applications in mechanical comminution and enforcing the reactivity of hard inorganic substances, mechanochemical treatment can be utilized as a “soft” chemical method (*chimie douce*) [61]. In such a scenario, mechanochemistry operates through molecular complexation [62], rather than impact. It is interesting to note that this view of soft mechanochemistry in the synthesis of inorganic materials appeared concomitantly with early studies of organic and supramolecular chemists on molecular recognition and complexation under grinding and milling [63].

## 4. Redox Reactions

Redox reactions are among the most abundant inorganic mechanochemical reactions. Typically, these involve either the oxidation of a metal or a semi-metal (metalloid) with a non-metallic element, such as oxygen, sulphur, or a halogen, or the reduction of a metal (or a metalloid) oxide, chalcogenide, or halide. Whereas the first reaction type has been of immense academic value in understanding the course of mechanochemical reactions, the latter reaction type is of considerable technological value, as it is related to metal extraction and purification.

### 4.1. Reactions of Elements and Compounds with Oxygen and Other Chalcogens

A number of metals, metalloids, and metal chalcogenides (sulphides, selenides) become pyrophoric upon mechanochemical milling treatment. The facile reaction with molecular oxygen is often highly exothermic and difficult to control. Consequently, the synthesis of oxides through the milling of raw elements in an oxygen-containing atmosphere is of little technological and synthetic importance. Furthermore, the increased activity towards oxygen of metals or metalloids finely dispersed by milling implies that great concern should be given to removing traces of air when such reactions are not desirable. On the other hand, it is also possible that parasitic reactions with traces of oxygen can affect the course of mechanochemical milling reactions, especially when milling sulphides, selenides, or hydrides. An interesting example of a metal reacting by being mechanically treated along with an impurity of oxygen gas is the milling of iron with ammonia at liquid nitrogen temperature. Due to the limitations of the experimental setup, air accidentally entered the chamber and the milling reaction produced FeO as a side-product, presumably as a result of the reaction between the metal and liquid O_2_ [64].

**Figure 2:**
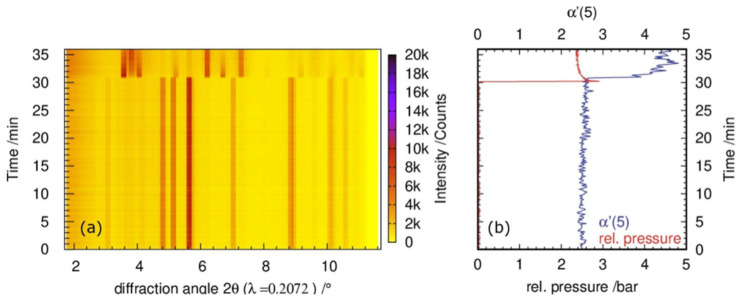
Real-time monitoring of the reaction of an equimolar mixture of zinc and sulphur being milled at a frequency of 27 Hz: (**a**) in situ PXRD synchrotron data and (**b**) pressure sensing data. The initial reaction time is confirmed using a calculated auto correlation parameter, α′(5) [67].

The direct synthesis of metal sulphides from sulphur and elemental metal precursors has been demonstrated for several materials of interest in energy and opto-electronic application [65,66,67]. In most cases, the transformations were found to be very rapid and sometime led to explosive reactions. For example, Baláž and co-workers have reported the ultrafast mechanosynthesis of copper sulphides by the ball milling of the elements, and the use of a customized planetary milling reaction vessel capable of either temperature and pressure sensing indicated temperatures as high as 950 °C (in case of CuS formation) or 700 °C (for Cu_2_S formation) at the moment of explosion [65]. Recently, the Weidenthaler group reported simultaneous in situ pressure and synchrotron powder X-ray diffraction (PXRD) during the monitoring of a ball milling reaction of elemental zinc and sulphur [67]. The in situ study revealed a sudden increase in pressure matching the sudden change of composition of the reaction mixture, initially forming the metastable wurtzite polymorph of ZnS, followed by the appearance of the thermodynamically preferred sphalerite phase (Figure 2).

#### 4.1.1. Mechanically Induced Self-Propagating Reactions (MSRs)

If the reaction between mechanically-activated reactants is sufficiently exothermic, the heat released at the onset of reaction might be sufficient to support the process to continue after mechanochemical treatment. In many cases of such a mechanochemically induced self-propagating reaction (MSR), the mixture ignites and the reaction further proceeds as a combustion process, also termed a self-sustained high-temperature synthesis (SHS). 

A practical guideline to predict whether a reaction will ignite into a MSR is if the ratio of reaction enthalpy (Δ*H*) and thermal capacity of the mixture (*C*) exceeds 2000 K. Owing to the peculiarities of mechanisms for each system investigated, however, this rule of thumb may not be reliable [68]. For example, mixtures of tin and selenium readily undergo MSR to form SnSe or SnSe_2_ although the Δ*H*/*C* value is around 1750 K, while the reaction of aluminium with sulphur does not ignite, despite the large Δ*H*/*C* value of 6400 K. 

Mechanically initiated self-propagating reactions are a well-studied sub-class of mechanochemical processes, which are of particular importance in inorganic transformations and the chemistry of oxide and sulphide minerals. Generally, a MSR is thought to proceed in three well-defined steps. The first step is the mechanical activation of the material, the so-called “miniforging” event in which ball-particle and interparticle collisions lead to the formation of solid solutions and may result in ignition. The second reaction step is the ignition after which the reaction becomes self-sustained. In most cases, the time for ignition ranges from seconds to minutes and, according to Takacs [68], is reproducible to 10% accuracy, allowing for the simple comparison of different MSRs. The third step of a MSR is mechanochemical milling after the self-propagating reaction has subsided, which ensures the completeness of the transformation and product homogeneity. The review by Takacs [68] provides an excellent overview of MSRs, highlighting a number of inorganic redox processes (e.g., reduction of oxides by metals, synthesis of chalcogenides, borides, carbides, silicides, halides, phosphides) combination reactions (e.g., the synthesis of oxo-anion salts, complex metal oxides and alloys), organic transformations (e.g., the reductions of organohalides with metal hydrides), as well as mathematical models applied to such reactivity. 

#### 4.1.2. Reactivity of Metal Oxides with Molecular Oxygen

The oxidation of mechanically activated binary compounds with molecular oxygen is generally less vigorous than in the case of pure elements. The strategy of ball milling, followed by exposure to oxygen gas was used to generate magnetic nanoparticles of chromium(IV) oxide (CrO_2_) [69]. Whereas bulk CrO_2_ is readily obtained by the oxidation of hydrated chromium(III) oxide, the ball milling of the starting material provided access to nano-sized precursor particles that retain their size even after oxidation. The synthesis of cerium(IV) oxide (CeO_2_) has been conducted mechanochemically by milling cerium(III) carbonate with sodium hydroxide in air [70]. The reaction presumably proceeds through the formation of an intermediate cerium(III) hydroxide phase. Cerium(III) hydroxide is further oxidized by air into hydrated cerium(IV) hydroxide that spontaneously crystallizes into CeO_2_·2H_2_O.

A much-studied example of a mechanochemical oxidation reaction involving a ternary inorganic compound with oxygen is the oxidation of the mineral ilmenite, FeTiO_3_, in air and in pure oxygen gas [71]. The milling of ilmenite is normally used to enhance the solubility of this mineral for further processing, for example in the production of titanium paint. It was established by Li and Liang [72] that milling of FeTiO_3_ in either air or in pure oxygen atmosphere results in the oxidation of Fe^II^ to Fe^III^ according to Equation (2):

6 FeTiO_3_(s) + 3/2 O_2_(g) → 2 Fe_2_Ti_3_O_9_(s) + Fe_2_O_3_(s) (2)

This mechanochemical oxidation was found to be sensitive to oxygen gas pressures below 27.4 kPa. For partial oxygen pressure above that value, the reaction rate was constant up to 152 kPa, indicating that the reaction is limited only by mechanochemical conditions and was zero order in oxygen. Consequently, it was possible to establish a mechanochemical reaction rate law (Equation (3)):

(3) $$-\frac{d\left(1-\alpha \right)}{dt}=2.1\times 10^{-5}R^{2.044}a_{c}^{2.616}{\left(1-\alpha \right)}^{2}$$

in which *α* is the fraction of product formed and the quantity $2.1\times 10^{-5}R^{2.044}a_{c}^{2.616}$ is the reaction rate. In the equation, *R* is the ratio of the weight of balls to the weight of milled sample and *a*_c_ is the centrifugal acceleration velocity, which must be proportional the milling frequency. The independence of the reaction rate for oxygen partial pressures over 27.6 kPa was explained by the saturation of oxygen binding sites on the surface of the milled sample.

**Figure 3:**
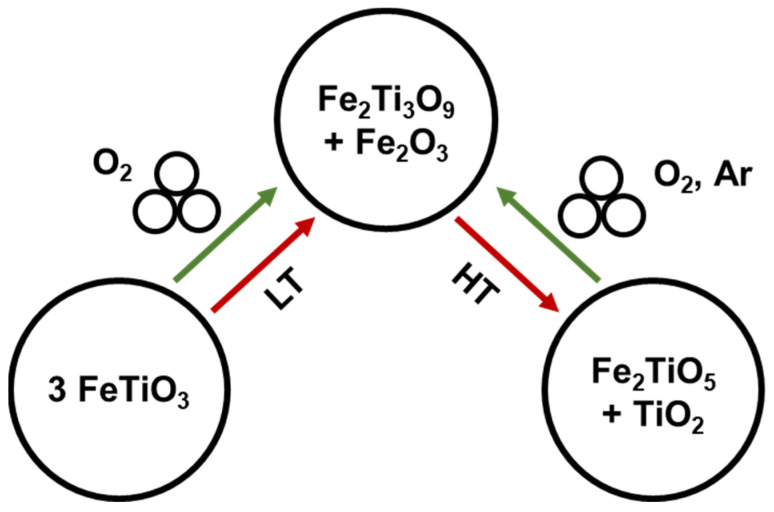
The mechanochemical ball milling (indicated by the three-circles symbol introduced by Rightmire and Hanusa) [28] and thermochemical low-temperature (LT) and high-temperature (HT) transformations of ilmenite. Adapted from [73].

The mechanochemical oxidation of ilmenite is different from the thermally conducted oxidation that takes place in two steps. Interestingly, mechanochemical milling at room temperature is capable of reversing the high-temperature step (Figure 3). The low temperature step is identical to the mechanochemical reaction, whereas the high temperature step involves the transformation of Fe_2_TiO_5_ according to Equations (4) and (5) [73]:

Fe_2_Ti_3_O_9_(s) → Fe_2_TiO_5_(s) + 2 TiO_2_(s) (4)

Fe_2_O_3_(s) + TiO_2_(s) → Fe_2_TiO_5_(s) (5)

#### 4.1.3. Reactivity with Peroxides, Peroxyacids, and Their Salts

As handling of oxygen gas remains technically demanding, alternative options have commonly been explored by synthetic chemist. Peroxides are commonly used, as aqueous solutions of hydrogen peroxide (H_2_O_2_), or in a solid form, such as sodium peroxide (Na_2_O_2_). The latter was used in the mechanochemical processing of molybdenum ore, mainly constituted of molybdenum sufide (MoS_2_), leading to the conversion of the ore into sodium molybdate (NaMoO_4_), corresponding to a formal oxidation from Mo(IV) to Mo(VI) [74]. Other examples are peroxy acids, such as peroxymonosulfuric (H_2_SO_5_) and peracetic acid (CH_3_CO_3_H) and their corresponding salts, which have now been demonstrated as suitable reagents for the mechanochemical activation of platinum group metals. Recently, Kravchuk and Forbes [75] used a 30% aqueous solution of H_2_O_2_ as a liquid-assisted grinding (LAG) [76] additive to enable the mechanochemical synthesis of uranium(VI) tri(peroxide) phases, such as Na_4_[UO_2_(O_2_)_3_]·9H_2_O, from the solid mixtures of UO_2_ and alkali metal peroxides. 

The platinum group and other noble metals, in particular gold, are known to barely react with O_2_ under normal conditions. This makes the use of extremely harsh conditions, with high temperature and corrosive reagents, such as chlorine gas or aqua regia, unavoidable in the industrial processing of such elements. As a means to address this technological challenge, Do and co-workers have reported a mechanochemical procedure using Oxone^®^, a bench-stable salt accessible on a large scale, comprising potassium peroxomonosulfate (KHSO_5_), potassium sulphate (K_2_SO_4_), and potassium hydrogensulfate (KHSO_4_), in a stoichiometric 1:0.5:0.5 ratio, as the oxidant. The milling of pure elemental metal in zirconia jars with three equivalents of Oxone and four equivalents of a halide salts (MX, where M = Na^+^, K^+^ or NH_4_^+^, and X = Cl^−^, Br^−^, I^−^) for 30–45 min was found to lead to the complete disappearance of the X-ray reflections of the metal in the PXRD pattern of the reaction mixture, and the formation of the targeted tetrahalometallate salts. Selectivity in reactivity was observed between Au, Pd, and Pt, opening the door to using such oxidative mechanochemistry as a strategy to separate and purify metals [77].

### 4.2. Synthesis of Nitrides: Reactions with Elementary Nitrogen vs. Reactions with Ammonia 

The conventional sources of nitrogen for nitride synthesis, a chemical transformation also known as *nitridification*, are nitrogen gas (N_2_) or ammonia (NH_3_). Owing to the exceptional stability of both of these molecules, it is clear that thermal metal nitride synthesis normally involves high temperatures. Due to the growing importance of nitrides as ceramic and refractory materials, it is also clear that the development of the low temperature methodologies of nitride formation is of considerable technological interest. Mechanochemical milling can allow for the simple preparation of transition metal nitrides directly from nitrogen gas, nominally at or near room temperature [78]. An example is provided by the mechanochemical synthesis of vanadium nitride (VN) by milling of vanadium metal under an increased pressure (11 atmospheres) of pure nitrogen. Within 8 h of milling, a sample of five grams of vanadium was completely transformed to the nitride, as evidenced by thermogravimetric analysis, electron microscopy, EELS, and powder X-ray diffraction. Generally, an elevated pressure of nitrogen gas was found to be beneficial for such mechanochemical nitride formation.

#### 4.2.1. Mechanisms of Nitride Formation with Nitrogen Gas

The synthesis of titanium nitride (TiN) represents an interesting process in terms of mechanistic complexity. The so far published data provide evidence that the reaction of Ti metal and N_2_ gas can proceed by two different mechanisms depending on the reaction conditions. Ogino and co-workers noted the formation of nitride phases TiN and (Ti, Al)N, respectively, through ball milling Ti and of titanium–aluminium alloys with nitrogen gas in a vibratory (shaker) mill [79]. The reaction is thought to proceed through the initial binding of nitrogen gas molecules onto a titanium surface, the dissociation of the resulting surface-bound nitrogen molecules into atoms, and the final dissolution of nitrogen atoms in the metal lattice and the formation of TiN. The rate of nitride formation was roughly proportional to the third power of the milling frequency, which was interpreted as evidence of the reaction taking place at hot spots formed at individual sites of ball impact. The facile transformation of titanium metal into TiN was confirmed by Criado and co-workers, who established 80% conversion by ball milling for 8 h under 1.4 atmosphere pressure of nitrogen in a swing mill [80]. The product was obtained as nanoparticles 10 nm in size. These authors also noted that the milling of titanium metal in argon and in nitrogen led to the formation of nanoparticles that exhibited enhanced ability to bind nitrogen gas even after milling. Consequently, the work of Ogino and co-workers, and of Criado and co-workers, indicated that the milling synthesis of TiN is based on the mechanical comminution of the metal into highly active nanoparticles (ca 9–10 nm in size), which readily form a surface TiN layer. The TiN layer prevents particle coalescence by further milling but allows the dissolution of nitrogen in the sample, therefore aiding reactivity. 

**Figure 4:**
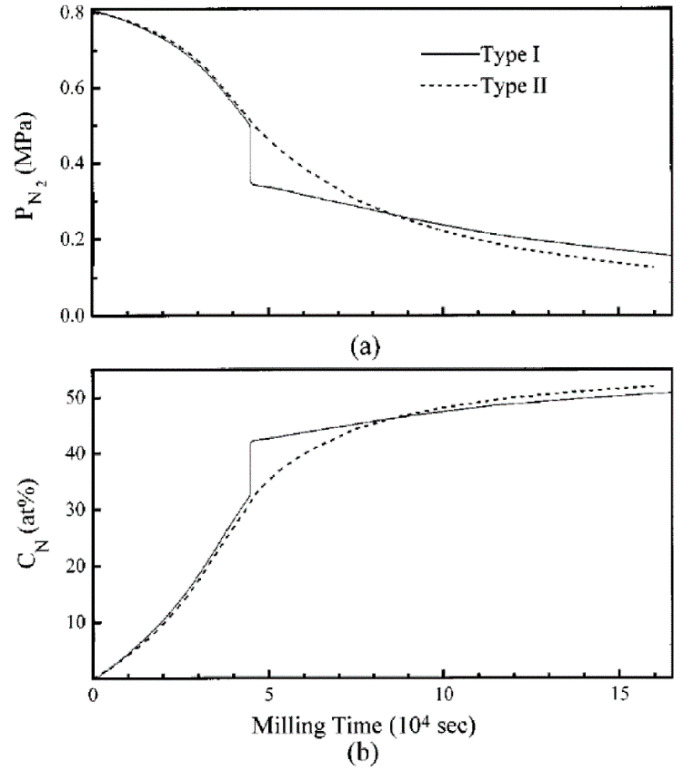
The difference between continuous formation and mechanically initiated self-combustion reaction (MSR) in the synthesis of TiN from the elements, illustrated by measuring: (**a**) the drop in nitrogen gas pressure during milling and (**b**) the change in nitrogen concentration within the milled solid sample [81].

In 1997, Chin and Perng established that the reaction of Ti metal and N_2_ can proceed either through a pathway involving either the continuous absorption of nitrogen gas, resembling reactivity described by other groups, or through a rapid self-combustion pathway, in which part of the nitrogen binding takes place in a rapid step, characterized by a sharp drop in nitrogen pressure within the milling chamber [81]. The second pathway is favoured by increasing the nitrogen pressure to over 6.5 atmospheres and by using harsher milling conditions. In particular, ball milling with steel balls weighing less than 2 g each led to continuous TiN formation, while balls heavier than 2 g generally led to a mechanically induced self-propagating reaction (MSR). The nature of the reaction was monitored continuously by measuring the drop of nitrogen pressure in the reaction vessel or by analysing the concentration of nitrogen in aliquots of samples milled for different times (Figure 4).

In 2002, the MSR of Ti metal and N_2_ in a planetary mill was confirmed by Gotor and co-workers, who established the complete conversion of reactants into TiN within 5 h [82]. For milling conducted at a pressure of 11 bars, powder X-ray diffraction analysis revealed the continuous formation of TiN until ca. 85 min milling. Between 85 and 94 min of milling, a sudden disappearance of the titanium metal reactant phase was observed. The in situ measurement of nitrogen gas pressure within the reaction vial indicated particle size reduction and the amorphization of titanium metal. Whereas these authors also noted a stepwise increase in the concentration of adsorbed nitrogen and the disappearance of titanium metal reflections from the sample powder X-ray diffraction pattern upon milling at 1.4 atmospheres of nitrogen, these changes are not as striking as for reactions under a high pressure of nitrogen gas. The synthesis of nitrides of niobium and tantalum, NbN and Ta_2_N, was reported by the milling of individual metals in nitrogen gas [83]. Whereas the formation of Ta_2_N was observed to take place within 16 h and in one step, followed by further dissolution of nitrogen in the product to yield an amorphous phase, the synthesis of NbN took place over 80 h and involved two intermediate crystalline phases, namely Nb_2_N and Nb_4_N_3_. The syntheses of TiN, NbN, and Ta_2_N illustrate three possible mechanisms for the mechanochemical synthesis of metal nitrides: (i) the formation of solid solution and its transformation into a crystalline nitride; (ii) self-sustained reaction; and (iii) the formation of intermediate crystalline nitride phases. Other mechanisms of nitride formation probably exist and, for example, the synthesis of vanadium nitride was reported to involve an initial polymorphic transformation of vanadium metal.

#### 4.2.2. Mechanisms of Nitride Formation with Ammonia Gas

Nitride syntheses generally proceed faster using ammonia rather than nitrogen gas. In some cases, the formation of the metal nitride is facilitated by the appearance of an intermediate hydride phase. Such stepwise reactivity was systematically explored for the synthesis of zirconium and titanium nitrides. The mechanochemical synthesis of iron nitrides from the metal and ammonia was systematically studied by Chen and co-workers [64]. Milling with ammonia at room temperature yielded Fe_3_N after ca. 400 h milling, whereas the product of milling with N_2_ was a high concentration solid solution of nitrogen in α-iron. The annealing of the solid solution at 500 °C leads to the formation of another nitride, γ-Fe_4_N. The synthesis of Fe_3_N, along with FeO due to experimental imperfection, leading to air being present in the reaction, was also achieved by milling Fe with solid ammonia at liquid nitrogen temperature. The formation of Fe_3_N at liquid nitrogen temperature was ten-fold faster than by milling at room temperature. If milling with either ammonia or nitrogen gas was conducted at 200 °C, the product was a solid solution of nitrogen in iron which, again, produces γ-Fe_4_N upon thermal annealing. The concentration of nitrogen in the solid solution made by milling at 200 °C was lower than that obtained by milling at room temperature. The improved dissolution of nitrogen in iron by milling at lower temperatures and the enhanced reactivity towards ammonia at liquid nitrogen temperature were explained by the greater efficiency of creating structural defects that promote nitrogen dissolution and diffusion in iron at low temperatures. Presumably, higher milling temperatures bring about the in situ annealing of mechanical defects and plastic deformations, therefore reducing the reactivity and concentration of dissolved nitrogen. In the synthesis of silicon nitride, Si_3_N_4_, the greater efficiency of ammonia as compared to N_2_ as the nitrogen source was explained by the ability of ammonia to remove inhibitory traces of oxygen from the surface of silicon by converting it into water [84]. This prevents the formation of a protective Si_2_N_2_O oxynitride layer and enables the full conversion of silicon into Si_3_N_4_. Consequently, the rationale for the use of ammonia instead of nitrogen in nitride synthesis would be the same as the use of graphite additive in the mechanosynthesis of metal hydrides (discussed below): the removal of an inhibitory oxygen-rich layer from the surface of the reactant.

### 4.3. The Synthesis of Phosphides from the Elements

The mechanosynthesis of phosphides directly from elemental metals and phosphorus has received significantly less attention than that of oxides or sulphides. The formation of nickel phosphide and of aluminium phosphide was systematically investigated by Takacs and Mandal [85], who found a wide variation of types of reactivity and a wide spectrum of products by increasing the phosphorus atom fraction *x* in mixtures of composition Ni_1−x_P_x_. By milling nickel metal and amorphous red phosphorus at low *x* values between 0.1 and 0.2, the authors observed either amorphization when using large milling media or crystallization to form tetragonal Ni_3_P when using smaller grinding media. The switching between amorphization and crystallization upon switching between ball charges was consistent with the behaviour previously established for the milling reaction of palladium and silicon. For *x* between 0.24 and 0.40 the milling reaction has a self-sustaining character of a mechanically induced self-propagating reaction (MSR), as the stoichiometric composition of the mixture now allows the formation of several highly exothermic nickel phosphide phases as major products: Ni_3_P, Ni_5_P_2_, and Ni_2_P with Δ*H*/*C* ratios between 2500 K and 2850 K. All three of these phases were observed in the milling of reaction mixtures with *x* between 0.25 and 0.40. At higher phosphorus contents, the mechanochemical reactivity again turned to gradual conversion, yielding Ni_5_P_4_ as the principal product at *x* = 0.5 and also NiP_3_ at *x* = 0.8. The formation of NiP_2_ was also observed which, surprisingly, appeared in a high-pressure cubic modification, as opposed to the room-temperature monoclinic one. The milling together of aluminium and red phosphorus in the 1:1 stoichiometric ratio readily yielded the cubic phase AlP, with a small amount of metallic iron observable in the powder diffraction pattern of the product after 5 h milling.

### 4.4. The Synthesis of Borides and Carbides

A number of borides are established in inorganic materials science as substances of high hardness and thermal stability. A notable example is rhenium diboride, ReB_2_, a super-hard and ultra-noncompressible ceramic material [86]. The traditional synthesis of ReB_2_ is conducted by directly combining the elements, complicated by the requirements for high temperatures above 2000 °C and the need for large excess (ca. 25%) of elemental boron which can significantly alter the properties of the product by forming a composite with ReB_2_. A mechanochemical synthesis has been demonstrated [87], providing a nominally room-temperature approach to this interesting ceramic. Although the extensive milling times (in excess of 60 h) led to the contamination of the product with tungsten and cobalt impurities, this mechanochemical reaction is highly attractive, as it enables the scalable synthesis of ReB_2_ directly from a 1:2 stoichiometric mixture of rhenium metal and boron, in air.

### 4.5. The Synthesis of Hydrides from Hydrogen Gas

The synthesis of binary (i.e., two-component) metal hydrides by reactive milling directly from the elements is illustrated by the work of Chen and Williams, who described the mechanochemical synthesis of hydrides TiH_1.9_, δ-ZrH_1.66_, and MgH_2_ from the native metals in a vertical planetary mill without any additives [88]. The quantitative formation of MgH_2_ and δ-ZrH_1.66_ was accomplished within 48 h, whereas TiH_1.9_ was obtained in 5.5 h. Of particular interest in materials science is the synthesis of magnesium hydride, β-MgH_2_, a potential hydrogen storage material thanks to its high hydrogen content and low cost of magnesium. Whereas the reaction between elemental magnesium metal and hydrogen gas proceeds only with difficulty under ambient conditions and hydrogen pressures between 5–11 atmospheres, the process can be improved using transition metal-based catalysts [89]. For example, Bobet and coworkers described that the reaction proceeds to ca. 35% upon 5 h milling without a catalyst, but in the presence of 10% cobalt metal as a catalyst over 70% yield of MgH_2_ is obtained in 10 h milling [90].

One of the principal obstacles to the synthesis of metal hydrides directly from the elements is thought to be the continuous regeneration of the natural oxide layer on the metal surface. To keep this natural oxide layer from regenerating, metals are typically first exposed to harsh temperature conditions. A potential alternative to such treatment is to mill the metal with a catalytic amount (ca. 10%) of graphite in an atmosphere of hydrogen. By using this technique, large improvements in room-temperature mechanochemical adsorption of hydrogen have been observed with Mg, Ti, V, Mg_2_Ni, and FeTi, leading to the complete (e.g., TiH_2_) or partial (e.g., HFeTi) formation of metal hydrides [91]. By combining this graphite-additive technique with increased temperature, the quantititive synthesis of β-MgH_2_ was accomplished within one hour, using 4 standard atmospheres of hydrogen gas at 300 °C. Without the graphite additive, the reaction produced only traces of β-MgH_2_ under the same conditions [92]. Extending the milling time to two hours led to the partial formation of another polymorphic form, the metastable γ-MgH_2_. Due to longer milling times, it might be that the formation of γ-MgH_2_ was enhanced by the adsorption of impurities from the stainless-steel milling assembly.

### 4.6. Synthesis of Ternary Compounds and Stoichiometric Control

A step further in the complexity of reaction mixtures is the synthesis of ternary (i.e., three-component) or higher-level compounds directly from their constituent elements. An example of such one-pot and multi-component mechanochemical synthesis is the formation of Sn_2_P_2_S_6_, prepared by the extended (80 h) low-energy milling of sulphur, phosphorus, and tin in air and in the appropriate stoichiometric ratio. The final product always contained a small amount of residual tin or residual tin and α-sulphur, as established by powder X-ray diffraction [93]. An excellent overview of the mechanochemical synthesis of ternary oxides has been given by Fuentes and Takacs [94].

It is often the case that binary, ternary and higher-order compounds exist in different stoichiometric compositions. When using conventional solution- or melt-based procedures, the selective preparation of such stoichiometrically distinct compounds can be hindered by kinetic or thermodynamic effects, e.g., due to the concomitant nucleation of stoichiometrically different compounds or through the preferred precipitation of the product that is least soluble. This makes it difficult or even impossible to selectively synthesize a particular stoichiometric variation of a compound starting from the exactly required ratio of constituents. This observation is valid not only in the context of inorganic syntheses but also in a general sense, across the areas of organic, metal–organic, and supramolecular synthesis (for example, cocrystallization). 

These limitations of synthetic chemistry in the liquid phase can be avoided in organic, metal–organic, and supramolecular synthesis by conducting the synthesis either by neat grinding or in the presence of a small, catalytic amount of a liquid phase (e.g., in liquid-assisted grinding). That the same is possible in the context of mechanosynthesis of inorganic compounds has been illustrated in different cases. For example, Shen and co-workers have conducted the selective mechanochemical syntheses of binary tin(II) and tin(IV) selenides, SnSe and SnSe_2_, by milling the constituent elements in the appropriate exact ratios [95]. In contrast, although there are two known ternary compounds of tin, antimony, and selenium, with compositions Sn_2_Sb_6_Se_11_ and SnSb_2_Se_4_, the milling of the three elements in suitable stoichiometric ratios always yielded materials isostructural to Sn_2_Sb_6_Se_11_. The result indicates the inability of this particular mechanosynthetic method to yield the known compound SnSb_2_Se_4_.

A class of materials that have gained considerable attention in recent years for application in photovoltaics and light-emission due to their fascinating photophysical properties are lead halide perovskites (LHPs), and their lead-free analogues [96,97]. These compounds have been shown to be accessible through simple one-pot multicomponent mechanochemical reactions between the suitable halides salts, including ammonium halide species for the hybrid materials of general formula RNH_2_PbX_3_ (where RNH_3_^+^ is an ammonium, formamidinium, or guanidinium cation) and excellent control of the photophysical properties of the synthesized product [98].

### 4.7. Unexplained and “Stochastic” Reactions

In many cases mechanochemical redox reactions can be explained by simply considering the thermodynamic reduction potentials of the chemical species involved. However, some exceptions arise simply through the complexity of the reaction mixture or due to the fact that mechanochemical reactions are sometimes dictated by non-equilibrium thermodynamics, or occur in a high-energy plasma-like environment. An example of a complex redox reaction is the reduction of copper(II) sulphate pentahydrate with iron, magnesium, and tin by toluene wet milling [99]. Whereas reduction in aqueous solution media typically leads to the formation of metallic copper, Varghese and co-workers found that the outcome of mechanochemical milling could not be straightforwardly related to the expected electrochemical driving force. Magnesium, which exhibited the highest difference of reduction potential with respect to copper(II), yielded copper(I) oxide, Cu_2_O, as the final product, In contrast, iron, with a lower difference of reduction potentials, affected the complete reduction of copper ions to metallic copper. The fastest reduction was observed for tin, which exhibited the smallest difference of reduction potentials and provided a mixture of copper and ß-bronze as the product. The unexpected behaviour of magnesium was explained through a more complex reaction mixture, involving a not yet identified intermediate, and the formation of a gas which was, tentatively, identified as hydrogen generated by a side-reaction between water and metallic magnesium.

Probably one of the most famous examples of surprising chemical reactivity obtained by mechanochemical means is the reduction of carbon dioxide with metallic gold to yield gold(III) oxide [100]. The electrochemical potentials indicate that the reaction is thermodynamically disfavoured by ca. 360 kJ mol^−1^, leading Thiessen and co-workers to explain this mechanochemical redox reaction gold through a non-predictable so-called “stochastic” nature of chemical processes taking place in a high-energy plasma environment generated by harsh milling. Whereas the gold reduction of CO_2_ is often cited, similar “inexplicable” reactions were reported in the early days of mechanochemical science, for example, the reduction of CO or CO_2_ by metallic copper (thermodynamically disfavoured by ca. 90 kJ mol^−1^) [101,102] or the reduction of sodium by the milling of sodium chloride with metallic mercury [103], according to Equation (6):

2 NaCl + Hg → HgCl_2_ + 2 Na (6)

Although such unusual chemical transformations have occupied much of the early reported mechanochemical and tribochemical work of Heinicke and Thiessen in 1960s and 1970s, the understanding of their underlying mechanisms has never reached a quantitative level. Consequently, “stochastic” chemical processes remain poorly understood and an open challenge to modern analytical and mechanochemical techniques. In particular, reactions involving reduction steps remain poorly understood, in particular those purely based on metallic species [104]. However, recent studies in the mechanochemical preparation of highly reducing species, such as electrides, might open the path to and increase understanding of these processes [105].

### 4.8. Reduction of Materials by the Metallic Milling Vessel

In the design and analysis of mechanochemical reactions, it is important to consider the possibility that the material from which the milling assembly is made could actively influence and/or participate in the course of a chemical transformation. Although there are known cases in which a mechanochemical reaction is catalysed by zirconia-based milling media [106], the participation of the milling assembly is much more established for metallic balls and/or vessels. For example, the milling of hydrated tetrachloroauric(III) acid in the presence of long-chain aliphatic amines in a steel-based milling assembly was unexpectedly found to lead to the formation of gold nanoparticles (AuNPs) [107]. The reaction was explained through the inadvertent galvanic reduction of the gold(III) precursors by the iron-based milling assembly. Although initially unexpected, this reaction was further developed into a targeted solventless strategy to synthesize amine-protected, size-controlled AuNPs, as the particle size was readily controlled in the 1–2 nm range through changing the length of the alkyl substituent on the amine. Another example of such an inadvertent transformation appears to be the reduction of uranium(IV, VI) oxides, including U_3_O_8_ and U_3_O_7_ to uranium(IV) oxide, UO_2_, reported by Kovacheva and co-workers [108]. The powder X-ray diffraction analysis of the reaction, which is conducted using a steel milling assembly, revealed the concomitant appearance of the X-ray reflections of UO_2_ and Fe_2_O_3_, indicating an active role of the milling vessel in the reduction of uranium(VI). 

While reactions involving the milling vessel require caution in the design of mechanochemical reactions, they also present a valuable and still not sufficiently explored route to simplify and control reactivity. The first inroads in that sense were made by the Mack group, the Borchardt group, as well as by others, by using the components of the metal-based milling assembly to catalyse organic transformations. Examples of such an approach are the use of an entirely copper-based milling assembly to conduct copper-free Sonogashira coupling reactions [109], the use of brass balls to catalyse the synthesis of the antidiabetic Tolbutamide [110], or the use of palladium-based balls to catalyse Suzuki coupling transformations [111].

## 5. Metathesis (Exchange) Reactions

Reactions of this type can be generally divided into displacements based on redox processes, acid–base chemistry, or the simple exchange of counterions between inorganic solids. The described mechanochemical reduction of copper(II) sulphate with metallic iron, as described above, is an example of a redox exchange reaction [99], while the reaction of barium carbonate with WO_3_ to give BaWO_4_ and CO_2_ is an example of an acid–base exchange. However, such reactions are more conveniently treated with other redox and acid–base reactions, so here we will focus exclusively on the reactions of exchange of counterions. An example of such reactivity is the reaction of a mixture of KCl and NaNO_3_ to form a mixture of KNO_3_ and NaCl. Among the most famous inorganic mechanochemical metathesis reactions are halide replacements that take place, for example, upon the mechanical compression of KBr tablets in infrared spectroscopy investigations. This phenomenon was systematically explored by Reguera and co-workers, who conducted the manual grinding (using an agate mortar and pestle) of ammonium, rubidium, and caesium hydrogendifluorides with potassium bromide [112]. In each case, the anion metathesis reaction took place to form KHF_2_ and the corresponding bromide, exemplified by Equation (7):

MHF_2_(s) + KBr(s) → KHF_2_(s) + MBr(s) (M^+^ = NH_4_^+^, Cs^+^, Rb^+^) (7)

Analogous reactivity was observed with sodium halides NaCl, NaBr, and NaI, following Equation (8):

NaX(s) + MHF_2_(s) → NaHF_2_(s) + MX(s) (M^+^ = NH_4_^+^, K^+^, Rb^+^, Cs^+^; X^−^ = Cl^−^, Br^−^, I^−^) (8)

All the reactions were tentatively rationalized by the increasing stability of the product hydrogendifluoride salt with the smaller cation. Following such reasoning, the exchange reactions with LiCl, LiBr, and LiI are expected to proceed most readily. Indeed, grinding with lithium halides readily provided the exchange products but also involved a side-reaction in which the HF_2_^−^ ion was transformed into F^−^ and gaseous HF (Equation (9):

MHF_2_(s) + LiX(s) → LiF(s) + MX(s) + HF(g) (M^+^ = Na^+^, K^+^, Rb^+^, Cs^+^, NH_4_^+^; X^−^ = Cl^−^, Br^−^, I^−^) (9)

The reaction is explained by the mechanochemical sensitivity of LiHF_2_ that, upon mechanical milling or upon heating, converts to the very stable LiF. Similar instability is demonstrated by NaHF_2_ upon longer grinding. Whereas powder X-ray diffraction was used for the qualitative analysis of the reaction mixtures, infrared spectroscopy enabled the quantitative assessment of the reaction yield. In that way, it was established that reaction rates for the exchange of caesium, rubidium, and ammonium hydrogenfluorides with KBr decrease in the sequence CsHF_2_ > RbHF_2_ > NH_4_HF_2_. The sequence was tentatively associated with the analogous decrease in hygroscopicity of the hydrogendifluoride reactants. keeping in mind that the reactions have been conducted by manual grinding, the infrared spectroscopy measurements yielded surprisingly well-shaped kinetic curves for these exchange reactions, possibly suggesting a first-order rate law.

More recently, Lukin and coworkers explored the ball-milling metathesis reaction between AgNO_3_ and NaX (X = Cl, Br, I) to form AgX and NaNO_3_ [113]. The reaction rates for milling reactions involving NaI and NaBr were similar and were significantly faster than those with NaCl, potentially due to the observed of the formation of the intermediate Ag_2_ClNO_3_, while the corresponding intermediates did not form with NaBr and NaI. To further understand the difference in observed kinetics, the authors prepared the Br and I intermediate, Ag_2_BrNO_3_ and Ag_2_INO_3_, respectively, through a slightly different mechanochemical reaction between AgX (X = Br, I) and AgNO_3_. Based on DFT calculated reaction enthalpy, the difference in reactivity is ascribed to faster reaction of AgNO_3_ with the introduced NaBr or NaI instead of the nascent AgX (X = Br, I).

As an alternative to the synthesis from elemental P discussed above, Fiss and coworkers reported the synthesis of ultrasmall Ni_2_P particles from anhydrous nickel(II) chloride (NiCl_2_) with sodium phosphide (Na_3_P) and long chain alkyl amine, used both as a reducing agent and to control the size of the final materials [114]. Similarly, simple and complex metal sulphide salts have been shown to be accessible through mixing reaction between suitable metal acetates and sodium sulphide (Na_2_S), such as the formation of ZnS and Cd_x_Zn_1-x_S from zinc and/or cadmium acetate [66].

## 6. Doping and Structural Rearrangements, Including Reactions with the Mechanochemical Reaction Assembly (Milling Jar and Balls)

The formation of mixed substances is of notable importance in the context of mechanochemical self-assembly. At the simplest level, this type of reaction involves grinding or milling together simple metal oxides to form mixed metal oxides belonging to a specific structural types. Of particular technological significance in this context are the structures of the spinel and perovskite type.

### 6.1. Doping with Inorganic Impurities from the Milling Vessel: Polymorphism

In contrast to the deliberate mechanochemical transformations of stoichiometric mixtures of binary substances into the materials of ternary or higher composition, mechanochemical milling can also lead to the often-accidental incorporation of atomic-level impurities into the final product. The inclusion of atom-level impurities into the lattice of milled material can result in significant changes to its properties and structure. This is well-illustrated in the study of the structural transformations of zirconium dioxide (zirconia, ZrO_2_) upon milling in stainless steel vessels [115]. Whereas the thermodynamically stable form of zirconia at ambient temperature and pressure is monoclinic (*m*-ZrO_2_), at temperatures between 1170 °C and 2370 °C, the material transforms into its tetragonal polymorph (*t*-ZrO_2_). The further heating of *t*-ZrO_2_ above 2370 °C results in the formation of cubic zirconia, *c*-ZrO_2_. It was, however, observed by Bailey et al. that ball milling of *m*-ZrO_2_ at nominally room temperature and in a metal milling assembly leads to the formation of metastable *t*-ZrO_2_ [116]. The unexpected transformation was initially ascribed to a surface energy effect. However, it was subsequently established that, if a zirconia-based milling assembly was used, the room-temperature formation of the metastable polymorphs was not observed. A combined study confirmed that the *m*-ZrO_2_→*t*-ZrO_2_ transformation indeed occurred in a stainless-steel milling assembly. The authors used PXRD to demonstrate that the transformation only took place after an induction time of milling of ca. 15 h and was completed after 20 h. At the same time, PXRD analysis also revealed a gradual increase in α-Fe content for up to 15 h, followed by its disappearance. These PXRD observations, accompanied by Mössbauer spectroscopy analysis, led Štefanić and co-workers to recognize that the formation and stabilization of *t*-ZrO_2_ at room temperature was a result of the incorporation of small amounts of Fe^3+^, Fe^2+^, or Cr^3+^ impurities, which were generated from the milling assembly based on hardened chromium steel. The application of Raman spectroscopy also indicated the further transformation of *t*-ZrO_2_ into *c*-ZrO_2_ at milling times of over 30 h. The ability to stabilize a metastable polymorph of zirconia through the incorporation of impurities represents an inorganic example that could be of relevance to the area of polymorph screening of molecular materials, which is of extreme importance in the context of pharmaceutical materials science [117]. In particular, although milling with impurities has not been inaugurated as a regular part of polymorph screening strategies for molecular materials, the recent study by Lancaster on the polymorphism of progesterone implies that the polymorphs of molecular crystals could also be stabilized by the incorporation of particular impurities [118].

### 6.2. Doping with Molecular Precursors: Photoactive Materials

Titania, TiO_2_, is one of the most promising inorganic photocatalysts for the photooxidation of organic substances owing to an electronic bandgap that allows exciton formation upon irradiation at ultraviolet (UV) and near-UV wavelengths. However, the efficiency of pure TiO_2_ as a photooxidation agent using sunlight is limited as the wavelengths compatible with titania-based photooxidation do not make up more than ca. 5% of the solar spectrum. The photoactivity of TiO_2_ is affected by its crystal structure, as the activity increases in the order brookite < rutile < anatase. Another means of improving the photoactivity of titania is by particle size reduction. It was demonstrated that nano-sized rutile particles exhibit a small bandgap of 3 eV, resulting in the better absorption of visible light and improved photocatalytic action compared to macroscopic anatase particles. However, the improvement of photoactivity by particle size reduction is of limited scope. Currently, the most promising strategy for enhancing the photocatalytic properties of titania is through the incorporation of nitrogen impurities [119]. The introduction of nitrogen is possible through sputtering a TiO_2_ target in dinitrogen gas [120], heating titania in NH3-containing atmosphere at 600 °C, or through a two-step room temperature solution process based on controlled hydrolysis and ammonolysis of TiCl_4_ [119], a simple mechanochemical alternative was proposed by Yin and co-workers [121]. These authors achieved mechanochemical nitrogen doping through the high-energy milling of P-25 titania (commercial titania composed of 75% anatase and 25% rutile forms) with hexamethylenetetramine as a nitrogen source. Whereas milling generally facilitates the transformation of anatase to the thermodynamically more stable rutile form, Yin et al. found that this transformation was slowed down by the presence of hexamethylenetetramine, probably through buffering the impact of grinding media [121]. The amount of nitrogen doping was up to 0.25% after three hours milling and, after calcination at 400 °C, the nitrogen-doped powder exhibited a yellow colour (absorption edges at 400 nm and 550 nm) and excellent activity in the photocatalytic removal of nitrogen monoxide using the visible light of the 510 nm wavelength. The inclusion of nitrogen was tentatively explained by the reaction of the oxide with ammonia, formed by the mechanochemical decomposition of hexamethylenetetramine, according to Equation (10):

C_6_H_12_N_4_ → 6 C + 4 NH_3_ (10)

The nature of the nitrogen dopant was initially described as a NO_2_^2−^ radical species [122] but was later re-assigned to a bulk radical N_b_ site [123]. Ammonium carbonate and urea were also used as an alternative source of ammonia in the milling reaction, again leading to the formation of highly active nitrogen-doped materials [124,125]. However, the activity of titania doped in this way was lower than with hexamethylenetetramine. Subsequent XPS studies indicated that hexamethylenetetramine also leads to the C-doping of TiO_2_, which could also be accomplished by the milling of P-25 titania with adamantane, C_10_H_16_ [126]. Despite a number of reports describing the enhanced photoactivity of mechanochemically nitrogen-doped P-25 titania, this property is still not fully understood: a recent report described very little improvement in photoactivity upon mechanochemical N-doping [127].

### 6.3. Energy Materials: Lithium-Graphite Intercalation Compounds

**Figure 5:**
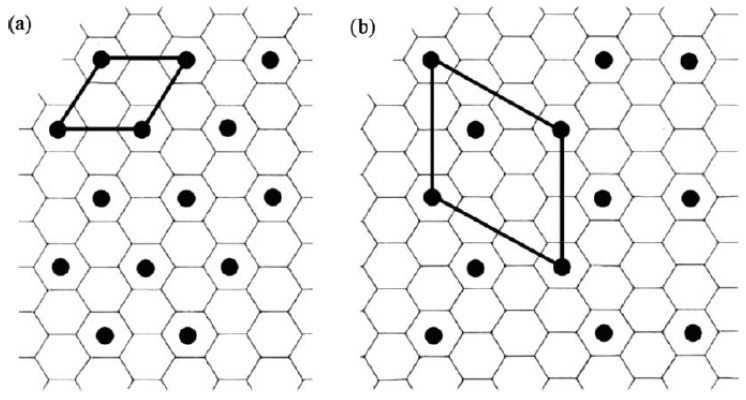
Representations of crystal structures of the lithium intercalation compounds with graphite: (**a**) first stage LiC_6_ and (**b**) second stage LiC_18_, projected perpendicular to graphite sheets. The honeycomb network represents an individual graphite sheet and black filled circles the positions of lithium ions. As each carbon atom is a part of three joined hexagons, it follows that each hexagon corresponds to two entire carbon atoms. Therefore, a formula such as LiC_6_ implies that there is only one lithium atom per three carbon hexagons. A superdense phase with a formula LiC_2_ would have one lithium atom intercalated per each hexagon.

Technologically highly important types of inorganic doped materials are graphite intercalation compounds. Lithium intercalates, in which lithium atoms are introduced between the sheets of graphite, are highly relevant as components of modern lithium-ion batteries (note that numerous metal oxides materials for lithium-ion batteries have been prepared through mechanochemical methods, as reviewed by Yang et al.) [128]. Whereas lithium intercalates formed through vapour-phase diffusion typically exhibit a “stage 1” composition LiC_6_ or a “stage 2” composition LiC_18_ (Figure 5), superdense phases with compositions LiC_3_ or LiC_2_, can be obtained under conditions of high temperature and stress. Such superdense phases are of great interest in modern energy storage industry. The ability to mechanochemically synthesize lithium graphite intercalates was described by Janot and Guérard, who systematically investigated the optimum reaction parameters for the mechanochemical production of superdense lithium-graphite materials [129].

In most cases, the direct milling of lithium and graphite powders provided the “stage 1” compound LiC_6_, even if higher lithium:carbon ratios are used. Importantly, in such cases, the excess lithium was not observable using powder X-ray diffraction, indicating either the complete amorphization or the formation of nanocrystalline particles that would be difficult to observe using this technique. Whereas the use of excess lithium did not yield higher stage phases than LiC_6_, it did accelerate the formation of LiC_2_ from 24 to 12 h.

**Figure 6:**
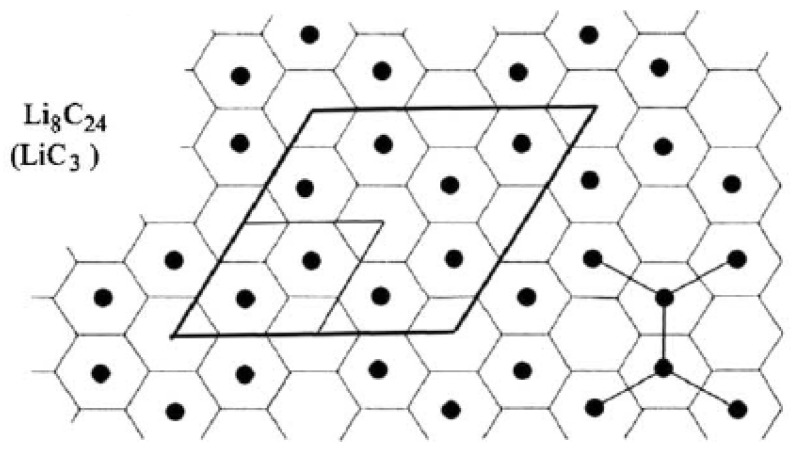
Schematic representation of the structure of a superdense lithium graphite intercalation compound LiC_3_, assigned through a combined X-ray powder diffraction, solid-state lithium NMR, and XPS study.

Attempts to improve the synthetic process by increasing the number of milling balls or increasing milling ball size did not succeed. In fact, the use of large radius milling balls (20 mm vs. conventional 5 mm) led to the complete absence of mechanochemical intercalation, illustrating that simply increasing the force of milling is not necessarily the best way of improving reactivity. Instead, conducting the milling process in the presence of an inert liquid, dodecane, was found to lead to the formation of a superdense phase of composition LiC_3_. The formation of LiC_3_ was observed when using 1 cm^3^ of dodecane per five grams of the solid reactant mixture, corresponding to the conventional liquid-assisted grinding (LAG) [130] conditions of *η* = 0.2 μL mg^−1^. Whereas the formation of LiC_3_ was first noted through density studies, a combined X-ray powder diffraction, XPS and solid-state Li NMR study provided a plausible structural model for this superdense graphite intercalation phase (Figure 6). The described formation of the LiC_3_ phase represents a clear illustration how the addition of substoichiometric amounts of a liquid phase can transform the course of a mechanochemical transformation in the context of inorganic synthesis.

### 6.4. Structural Rearrangements and Mechanochemical Activation of Complex Oxides 

**Figure 7:**
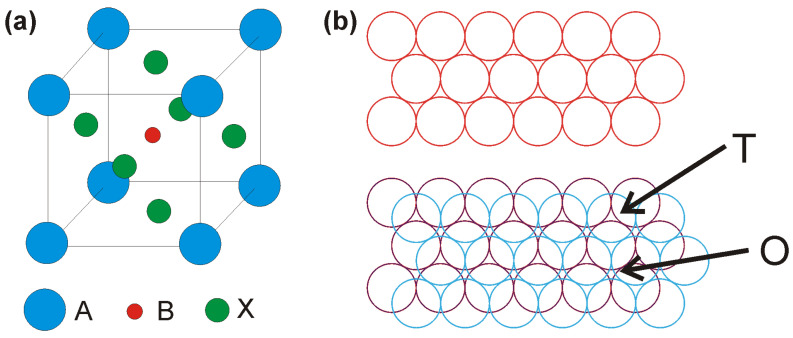
Schematic representations of notable inorganic structure types: (**a**) the spinel structure of a compound with composition ABX_3_ and (**b**) the spinel structure consisting of layers of hexagonally arranged anions (top, schematic representation of a single layer in red) with cations located in the octahedral (O) or tetrahedral (T) holes formed between adjacent layers (bottom, schematic depiction of T and O sites between two layers, upper layer in blue).

A well-known effect in mechanochemical processing is the introduction of structural defects in the structures of complex metal oxides. Particularly well-studied have been the processes of mechanical activation of spinel materials, such as spinel ferrites, and this area has recently been reviewed by Šepelák and co-workers [131]. Among the best studied systems is zinc ferrite, ZnFe_2_O_4_, a popular material for the removal of H_2_S from industrial gases. Zinc ferrite adopts a normal spinel structure, which means that the trivalent (Fe^3+^) occupy almost exclusively the octahedral sites in the close-packed lattice of anions (O^2−^) ions, whereas the divalent ions (Zn^2+^) occupy the tetrahedral sites of the oxide lattice (Figure 7). The octahedral and tetrahedral sites in a spinel structure are very often designated as ‘A’ and ‘B’ sites, respectively. Most spinel structures exhibit a certain degree of structural inversion, which is interpreted by a fraction of trivalent ions found in the tetrahedral (A) site. This structural inversion is typically designated as λ, leading to the general formulation of a spinel structure as (M^II^_1-λ_M^III^_λ_)(M^II^_λ_M^III^_2-λ_)O_4_ where M^II^ and M^III^ correspond to the divalent and trivalent cationic species, respectively. It was established by Tkáčová and co-workers that the mechanical milling of zinc ferrite samples leads to their activation, in the form of a structural inversion of the spinel structure [132]. Specifically, mechanical milling brings about the partial transfer of Fe^3+^ ions from octahedral to tetrahedral spinel structure sites [133,134]. Such structural inversion, as was established using 2 g samples of starting material, can populate about 94% of the available (and normally forbidden) tetrahedral sites with Fe^3+^ ions in less than half an hour (within 24 min). It has been calculated that the energy difference between the normal and completely inverted zinc ferrite spinel structures amounts to roughly 40–50 kJ mol^−1^, providing a rough approximation of the energies contained in a mechanically activated material. The speed of mechanical activation by milling is illustrated by the fact that the degree of inversion corresponding to a statistical distribution of cations (67% or 2/3 as the spinel structure exhibits twice as many tetrahedral than octahedral sites) is reached after only 5 min of milling. The mechanical activation of zinc ferrite strongly affected its stability, as evidenced by the observation that the annealing of the less activated samples, prepared by 5 and 12 min of milling above 330 °C, led to a thermal re-crystallization and the full recovery of the initial spinel structure. However, samples ground for 24 min exhibited partial decomposition into a mixture of zinc and ferric oxides above 500 °C, i.e., at a temperature that is ca. 700 °C below that at which ZnFe_2_O_4_ normally forms from individual oxides [132]. The mechanochemical activation of another normal spinel structure, CdFe_2_O_4_, demonstrated similar behaviour [135]. In contrast, the mechanical activation of the inverse spinel structures of magnesium ferrite (MgFe_2_O_4_) and nickel(II) ferrite (NiFe_2_O_4_) consists of the transfer of Fe^3+^ ions from tetrahedral to octahedral sites, again resulting in a structural disorder and almost statistical distribution (degree of inversion 0.73 and 0.72 for magnesium and nickel(II) ferrites, respectively) of cations over the available tetrahedral and octahedral sites [134,136]. The randomization of cation distribution over spinel A and B sites upon mechanochemical activation is not limited only to ferrite materials. The use of high-resolution ^27^Al solid-state NMR spectroscopy and transmission electron microscopy demonstrated that the mechanical activation of the normal spinel ZnAl_2_O_4_, partly inverse spinel MgAl_2_O_4_, and fully inverse spinel Li_0.5_Al_2.5_O_4_ involves the formation of a crystalline core-amorphous shell nanoparticle structure as well as the statistical re-distribution of cations across the tetrahedral and octahedral sites [137].

A different type of structural activation was observed in the mechanical milling of Bi_2_Ga_2_Al_2_O_9_, a complex oxide of the mullite-type crystal structure [138]. Whereas the mullite structure also provides a choice between octahedral and tetrahedral sites for trivalent metal cations (e.g., Al^3+^ or Ga^3+^), solid-state ^27^Al NMR studies on the milled samples of this material have shown no evidence of cation transfer between these sites. However, extended milling led to the formation of defects in the form of low-coordination aluminium ions with three or five oxide ligand neighbours. The formation of these low-coordination species was enhanced at high milling times and was concomitant with the reduction in particle size. For mechanically prepared nanoparticles of Bi_2_Ga_2_Al_2_O_9_, with a diameter of roughly 10 nm, the amount of such low-coordination defects can be as high as 21%. The current explanation for this effect is found in a high surface-to-volume ratio of nanoparticles, which leads to a large fraction of material being found in the form of partially disordered surface species.

## 7. Acid–Base Reactions: Synthesis of Mixed Metal Oxides

The formation of mixed metal oxides is a topic of particular technological relevance, due to a number of unique applications for such materials in the energy and electronic industry as electrode materials, memory storage devices, detectors, phosphors, semiconductors, or superconductors. The applications of such materials are often associated with a particular spatial distribution of metal cations in a relatively small number of structural types, of which the most notable are the perovskite and spinel structures. The perovskite type structure is often adopted by compounds with chemical composition ABX_3_, where A and B are usually metal cations of fairly different sizes and X is an anionic species, such as oxide (O^2−^) or fluoride (F^−^). This structural type (Figure 7a), which was named after the mineral Perovskite (CaTiO_3_, calcium titanate), is best described as a primitive cubic arrangement of the larger cationic species (A), in which the smaller cations (B) adopt a central position. The anions (X) are located in the centres of cube faces. The ideal, highly symmetrical cubic structure is readily distorted by variations in the size and nature of component ions, leading to less symmetrical and even dynamic structures that are of paramount importance in the understanding and design of modern magnetic and electrical materials.

The spinel structure (Figure 7b) is adopted by compounds with stoichiometric composition AB_2_O_4_, where A is a divalent and B is a trivalent cation. The spinel structure is one of the central ionic structure types of inorganic structural chemistry and is best described as a close hexagonal packing (hcp) of anions, with A and B cations occupying the tetrahedral and octahedral holes formed in the structure between neighbouring anion layers. In a normal spinel oxide structure, the divalent cations (A) largely occupy the tetrahedral holes, while the trivalent cations (B) are largely located in the octahedral holes. In an inverted spinel structure, all or some of the A cations are located in octahedral holes, while the B cations occupy the octahedral, as well as tetrahedral sites. For cations of transition metals, such as Fe^3+^, Mn^2+^, or Ni^2+^, switching between octahedral and tetrahedral environments can strongly influence the intrinsic magnetic and electrical properties of the material. Hence, the ability to mechanochemically synthesize normal or inverse spinel structures in a controlled manner is of considerable technological importance.

### 7.1. Synthesis of Normal Spinel Ferrites

The diffusion-controlled solid-state transformation of a mixture of ZnO and Fe_2_O_3_ into zinc ferrite at high temperatures has been studied by a number of groups, for example, by Toolenaar and by Halikia and Milona [139,140]. These groups explored the reaction kinetics between 600 °C and 800 °C. The reaction of ZnO and Fe_2_O_3_ under explosive pressure is sometimes cited as the first report of a mechanochemical synthesis of a ferrite material. The room-temperature transformation of ZnO and Fe_2_O_3_ into zinc ferrite by mechanochemical milling was demonstrated by Lefelshtel and co-workers, who monitored the reaction by Mössbauer spectroscopy and X-ray diffraction [141]. Mössbauer spectra indicated the appearance of zinc ferrite after 240 h of milling, and after 540 h its yield was no more than 32%. The same authors also conducted a reaction between zinc carbonate (with a formula given as ZnCO_3_) and α-Fe_2_O_3_ and observed ferrite formation after 100 h milling. Interestingly, the decomposition of the carbonate was observed significantly earlier (50 h of milling) than the formation of zinc ferrite, without any ZnO observable by powder X-ray diffraction. The authors have explained this through the formation of ZnO in the form of very small crystallites, which are difficult to detect by diffraction methods. However, a related and alternative explanation would be through the formation of a non-crystalline amorphous zinc-containing phase. This explanation is supported by the subsequent studies by Kim and Saito [142], who obtained the complete conversion of the 1:1 reaction mixture of ZnO and Fe_2_O_3_ within 4 h. The difference between reaction rates observed by Lefelshtel and co-workers [141] and Kim and Saito [142] is most likely related to the significantly smaller reaction scale used by the latter group (4 g). However, they also observed the complete disappearance of ZnO reflections from the PXRD pattern of the reaction mixture after only one hour milling. This observation is consistent with the reaction involving an activated, non-crystalline zinc-containing phase that would be invisible to X-ray diffraction techniques. The first weak X-ray reflections of the ZnFe_2_O_4_ product were observable in the diffraction pattern of the milled material only after 2 h of milling, further indicating the existence of an amorphous, zinc-rich intermediate phase. Subsequent X-ray photoelectron studies have revealed an unexpectedly large population of Zn^2+^ cations in the octahedral sites of the spinel structure, demonstrating an important structural difference between the mechanochemically synthesized ZnFe_2_O_4_ and the ordered material synthesized by conventional high-temperature processes. The structural disorder consisting in the increasingly statistical distribution of metal cations between the octahedral and tetrahedral sites of the spinel structure was confirmed by Goya and Rechenberg by magnetic susceptibility and the Mössbauer spectroscopy of the mechanochemically prepared zinc ferrite [143]. These authors proposed that structural disorder of the mechanochemical product is the result of a so-called “core–shell” nanoparticle structure in which the crystalline and ordered nanoparticle core is encased in a disordered amorphous shell that acts as a spin glass. Consequently, the nature of the mechanochemically synthesized zinc ferrite resembles that for the mechanically activated material, with a large degree of structural inversion and a crystalline core–amorphous shell structure of particles.

### 7.2. Synthesis of Inverse Spinel Ferrites

In contrast to ZnFe_2_O_4_, the analogous magnesium analogue MgFe_2_O_4_ adopts an inverted spinel structure with almost all trivalent cations in the tetrahedral sites. However, when mechanochemically activated, or when obtained by high-speed ball milling, MgFe_2_O_4_ exhibits an almost completely statistical (67%) distribution of trivalent cations in the tetrahedral sites [144]. Nickel(II) ferrite, NiFe_2_O_4_, is a related ferrite material whose mechanochemical synthesis was first attempted by mechanochemical route by Lefelstehl and co-workers but without success. Nickel(II) ferrite adopts an inverted spinel structure in its native bulk state. Whereas Lefelshtel’s initial synthetic attempts were unsuccessful, Jovalekić and co-workers subsequently achieved a relatively rapid synthesis of NiFe_2_O_4_ from a mixture of NiO and Fe_2_O_3_ within 35 h [145]. The synthesis was followed very recently by an even shorter one, which reduced the synthesis time down to 8 h [146]. Again, the difference in reactivities between the original experiments reported by Lefelshtel and the later investigators can be tentatively explained by the reduction of sample size to 30 g accompanied with a relative increase in milling vessel volume (500 cm^3^) and a larger milling ball-to-sample weight ratio (20:1). The monitoring of this mechanochemical synthesis using powder X-ray diffraction disclosed an important difference from the analogous process with ZnO: the X-ray reflections of both NiO and Fe_2_O_3_ reactants persisted in the reaction mixture for up to 20 h of milling. The mechanochemical formation could also be readily followed by studying the magnetization properties of the milled reaction mixtures. A sudden jump in magnetization occurred in samples milled between 20 and 35 h, indicating the intense formation of the nickel(II) ferrite phase. In contrast, the magnetization properties of the nickel oxide reactant milled on its own exhibited virtually no change, indicating that the incorporation of metallic iron from the milling assembly has little or no influence on the mechanosynthesis of NiFe_2_O_4_. The nanocrystalline structure of the mechanochemically synthesized nickel(II) ferrite was investigated by Šepelak and co-workers by a combination of ^57^Fe Mössbauer spectroscopy, magnetization studies, powder X-ray diffraction and TEM analysis [146]. This extensive and multi-faceted study yielded a detailed picture of the structural ordering in NiFe_2_O_4_ nanoparticles (6–13 nm in size) synthesized by milling. The study revealed the formation of spherical nanoparticles with a core-shell structure, in which the core is composed of a highly magnetically ordered inverted spinel structure, as expected for NiFe_2_O_4_, whereas the ca. 1 nm thick shell contains magnetically disordered cations that are randomly distributed between octahedral and tetrahedral sites.

### 7.3. Particle Structure of Mechanochemically Synthesized Mixed Metal Oxides

**Figure 8:**
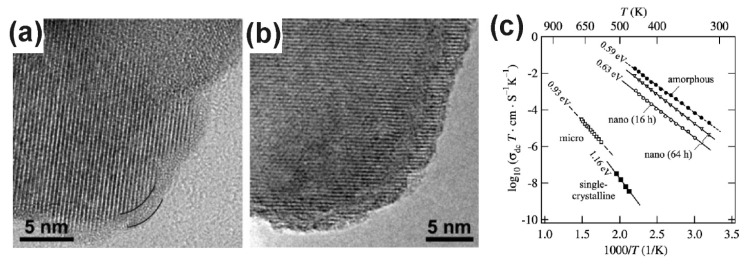
(**a**) Transmission electron microscopy image of the core–shell structure of the LiNbO_3_ ion conductor nanoparticle prepared by mechanochemical milling; (**b**) the core–shell structure is much less evident in a sample of LiNbO_3_ prepared by solution methods. (**c**) The comparison of conductivity (σ) for different forms of LiNbO_3_, including the amorphous materials and single crystals, as well as nanoparticles prepared by 16 h and 64 h milling [147].

A number of detailed investigations, largely conducted by Šepelák and Becker, involving transmission electron microscopy, solid-state NMR, and low-temperature Mössbauer spectroscopy [147,148,149,150,151], have demonstrated that the non-equilibrium distribution of cations between tetrahedral and octahedral sites is a general property of mechanochemically synthesized and mechanically activated spinels. Moreover, it is very likely that a crystalline core–amorphous shell structure is a general feature of mechanochemically synthesized or mechanically activated complex metal oxides, and that it plays a decisive role in controlling their unusual properties. For example, the enhanced lithium ion conductivity (*σ*) of the mechanochemically prepared perovskite LiNbO_3_ nanoparticles was explained through the presence of an amorphous grain boundary, observed by a combination of techniques, including transmission electron microscopy and EXAFS measurements (Figure 8) [147]. A similar nanoparticle structure, involving a 2 nanometre-thick amorphous shell associated with a 11–30 nanometre particle, was used to rationalize the enhanced room-temperature conductivity of the complex fluoride with a perovskite structure, LiBaF_3_, formed by milling together LiF and BaF_2_ [149]. The core–shell structure of the distorted perovskite ferrite BiFeO_3_ was described by Da Silva and coworkers [150]. Beyond the spinel structures composed of divalent and trivalent ions, the core–shell structure, the distortion of octahedral coordination geometry, and the non-equilibrium distribution of cations have been also observed for an inverse spinel stannate, Zn_2_SnO_4_, composed of divalent and tetravalent cationic species [151]. The structural deformation and core–shell structure have also been observed in mechanochemically prepared calcium stannate, Ca_2_SnO_4_, in an excellent example of a multi-faceted instrumental study of mechanochemical reactivity. As evidenced by a combination of powder X-ray diffraction, electron microscopy, ^119^Sn Mössbauer spectroscopy and ^119^Sn solid-state NMR spectroscopy, the milling of SnO_2_ and CaO in a 1:2 respective stoichiometric ratio yields Ca_2_SnO_4_ in 93% purity along with 7% perovskite structure CaSnO_3_. In contrast to previously mentioned examples, the disordered octahedral environment of the tin(IV) cation in Ca_2_SnO_4_ is more symmetrical than in the bulk crystalline solid [148].

### 7.4. Alternative Mechanochemical Procedures for the Synthesis of Mixed Metal Oxides

Mechanochemical milling in combination with thermal annealing has been successfully applied for the synthesis of a wide range of spinel and perovskite structures from a diversity of starting materials. The mechanochemical milling of MgO and Al_2_O_3_ readily provides the spinel MgAl_2_O_4_, whereas annealing a milled mixture of MgO and TiO_2_ yields either the perovskite structure Mg_2_TiO_4_ by brief annealing at >1000 °C or the thermodynamically more stable perovskite MgTiO_3_ after long annealing at a high temperature [152,153]. The mechanochemical treatment of a mixture of Mg(OH)_2_ and Al(OH)_3_ leads to complete amorphization after 120 min of milling, followed by the slow crystallization of a partly dehydrated Boehmite phase AlO(OH) after 240 min of milling. However, the calcination of the milled amorphous mixture above 800 °C leads to the rapid formation of a spinel structure MgAl_2_O_4_ [154]. Manganese(II) ferrite spinel has been obtained by the thermal annealing of a magnetite structure, MnFe_2_O_4_, synthesized by the reductive milling of Mn_2_O_3_ and Fe_2_O_3_ either in argon or in the presence of manganese metal [155]. Whereas the authors explained the formation of MnFe_2_O_4_ from Mn_2_O_3_ through a redox reaction involving the liberation of oxygen gas, it is not clear why another pathway, in which Mn^III^ would be reduced by the metallic milling assembly, would not be possible. Indeed, the milling of metallic iron with hematite, Fe_2_O_3_, led to the initial formation of a magnetite phase, Fe_3_O_4_, that was subsequently completely reduced into Wüstite, FeO [156]. An alternative synthesis of spinel ferrites has been explored by Ding and co-workers by the mechanical alloying of magnetite Fe_3_O_4_ and Co_3_O_4_ in relative ratios 2:1 and 2.2:0.8 [157,158]. In both cases, the product was a mixed magnetite phase of composition CoFe_2_O_4_ or Co_0.8_Fe_2.2_O_4_ that could be converted into the desired spinel ferrite only upon annealing above 700 °C. Mechanochemical milling has also been explored for the synthesis of mixed metal spinel ferrites. The milling of ZnO, MnO, and Fe_2_O_3_ led to the facile formation of the mixed ferrite (Mn,Zn)Fe_2_O_4_ with a non-equilibrium distribution of cations over tetrahedral and octahedral sites. The ordering of this mechanochemically synthesized phase was achieved by thermal annealing. A comparative study of mechanosynthesis and mild solution synthesis (“*chimie douce*”) has been performed on Fe_2.5_Ti_0.5_O_4_, a titanoferrite material exhibiting an inverted spinel structure. The synthesis was reported by Guigue-Millot and coworkers [159], who optimized the energy input in the mechanochemical process so as to achieve quantitative synthesis within 4 h of continuous grinding in a planetary mill with steel milling tools. Combined surface area measurements, scanning electron microscopy, and HRTEM analyses revealed the mechanochemical product to be a powder composed of particles with a broad size distribution and a mean size of 12 mm. The broad range of particle sizes was used to explain the significant differences observed between predicted and measured magnetic properties of the material. Overall, the particle size distribution and the resulting magnetic properties of Fe_2.5_Ti_0.5_O_4_ were found to be much more satisfactory if produced by a solution synthesis route. The syntheses and properties of a large number of mechanochemically prepared spinels and perovskites as well as other mixed oxide ferroelectric phases have recently been reviewed by Kong and co-workers [160] and by Šepelák and co-workers [138].

### 7.5. Synthesis of Oxides by Manual Grinding

Whereas the synthesis of stannates, ferrites, and associated materials typically requires conditions of high-speed milling over extended periods of time, a much milder form of mechanochemical reactivity was observed for the formation of oxo-salts of lithium [161]. The simple manual grinding of hydrated lithium hydroxide (LiOH·H_2_O) with molybdenum(VI) oxide in a mortar and pestle led to the formation of lithium molybdate according to the acid–base reaction (Equation (11)):

2 LiOH·H_2_O + MoO_3_ → Li_2_MoO_4_ + 3 H_2_O (11)

Importantly, the reaction was not observed when anhydrous lithium hydroxide was used, indicating that the presence of water in the solid reactant was critical. Analogous reactivity was utilized to synthesize an ionic conducting material belonging to the family of layered perovskite-type structures known as Ruddlesden–Popper phases [162]. In particular, when the perovskite-like solid acid HLaTiO_4_ (obtained through the reaction of the isostructural solid NaLaTiO_4_ with aqueous hydrochloric acid) was reacted with one equivalent of LiOH·H_2_O by grinding for 30 min using a mortar and pestle at ambient temperature. The reaction mixture did not become noticeably sticky or wet, powder X-ray diffraction analysis revealed that the material, while retaining isostructurality to the solid acid HLaTiO_4_, also underwent a noticeable contraction of the crystallographic parameters consistent with the formation of LiLaTiO_4_. The investigation of the as-prepared particles of LiLaTiO_4_ via transmission electron microscopy revealed monolithic particles resembling those of the starting material HLaTiO_4_, which was interpreted as an indication of the transformation taking place via a solvent-free H^+^-Li^+^ ion exchange process rather than a water-mediated dissolution-recrystallization process. The solid-state reaction could also be readily performed with sub-stoichiometric amounts of lithium hydroxide, leading to a family of isostructural Ruddlesden–Popper phases with compositions H_1−x_Li_x_LaTiO_4_, which exhibited a continuum of crystallographic lattice parameters between those corresponding to pure LiLaTiO_4_ and HLaTiO_4_. The subsequent thermal dehydration of these hybrid materials led to the formation of materials with the composition Li_x_LaTiO_4−x/2_, of which Li_0.5_LaTiO_3.75_ exhibited ion transport properties resembling those of the LiLaTiO_4_ end member of the series. Consequently, the manual grinding reaction, followed by thermal treatment (when the sub-stoichiometric amounts of LiOH·H_2_O are used) depicts a complete solvent-free approach for the synthesis of new on conductors.

It is worth highlighting a potential parallel between the above-described synthesis of lithium molybdate and the synthesis of model pharmaceutical cocrystals, as illustrated by the reaction of caffeine or theophylline with citric acid. Although comprising very distinct compositions, both reaction systems readily react through mechanical action if at least one of the reactants is present in the form of a hydrate [163]. Although the precise mechanisms through which the presence of the water of crystallization can assist in such nominally solvent-free processes is not known, the ability to draw a parallel between mechanosyntheses involving purely inorganic and purely organic compounds is striking.

The ability to synthesize inorganic materials by simple manual grinding also extends beyond compounds based on lithium oxide. For example, the manual grinding of a mixture of aluminium and magnesium nitrates with sodium hydroxide has been reported by Ay and coworkers to generate members of an important family of inorganic layered clay-like materials, known as layered double hydroxides (LDHs) [164].

**Figure 9:**
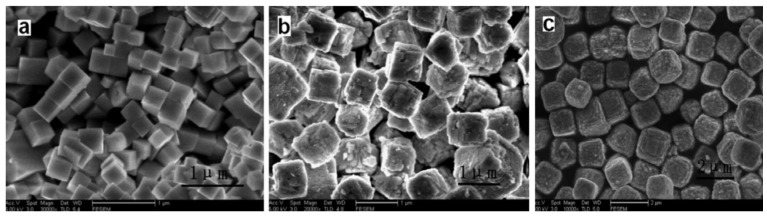
Particle size evolution in the manual grinding synthesis of zinc hydroxystannate. Scanning electron microscopy images of the product ZnSn(OH)_6_ after: (**a**) 10 min; (**b**) 30 min, and (**c**) 1 h grinding [165].

The synthesis of a perovskite-structured complex metal hydroxide by manual grinding in air was reported by Wang and co-workers, who conducted a one-pot three-component grinding reaction of zinc acetate dihydrate, tin(IV) chloride pentahydrate, and sodium hydroxide [165]. Within 10 min manual grinding, the powder X-ray diffraction pattern indicated the complete conversion into zinc hydroxystannate, ZnSn(OH)_6_. Surprisingly, the grinding reaction yielded the product as uniform cube-shaped crystals with ca. 350 nm edge. Grinding for more than 10 min led to the increase of crystallite size and surface roughening, so that 1.8 μm edge crystals were obtained after 60 min manual grinding (Figure 9).

## 8. Mixed Reactions

### 8.1. Solvent-Free Zeolite Synthesis and Structure Templating

In addition to close-packed oxide materials, of immense technological importance are also the related porous structures of zeolites. Zeolitic materials have found applications in gas cracking and separation, catalysis, washing powders, and chemical processing, and they represent an industry whose worth was assessed at ca. 50 billion dollars annually worldwide [166]. However, in contrast to mixed and doped metal oxides, the mechanosynthesis of open zeolitic structures is largely underdeveloped. To the best of our knowledge, the first mechanochemical process, giving rise to a zeolite structure, was reported in 2003 by Gordina and co-workers who milled a mixture of hydrated sodium silicate (Na_2_SiO_3_·6H_2_O), hydrargillite (Al(OH)_3_) and hydrated silica gel [167]. The analysis of the reaction mixture after 30 min milling using powder X-ray diffraction indicated the formation of the porous zeolite NaA structure. The reaction was brought to completion in a thermal calcination step at 450 °C. The synthesis of zeolite NaA from alumosilicates was reported in 2012 by the same group, who explored a sequence of mechanical milling and calcination from three types of reactions (Equations (12)–(14)) [168]:

6 (Al_2_Si_2_O_7_·2H_2_O) (kaoline) + 12 NaOH → 12 NaAlSiO_4_ (nepheline) (12)

6 (Al_2_Si_2_O_7_) (metakaolin) + 12 NaOH → Na_8_Al_6_Si_6_O_25_ (sodalite) + other feldspathoids (13)

6 (Al_2_Si_2_O_7_) (metakaolin) + 12 NaOH + 3 γ-Al_2_O_3_ → Na_12_Al_12_Si_12_O_48_ (zeolite NaA) (14)

Attempts to synthesize zeolite NaA from kaolin or metakaolin (Equations (12) and (13)) all led to the formation of nepheline or sodalite structures. However, the use sodium hydroxide and γ-alumina as an in situ source of sodium aluminate, based on tetrahedral AlO_4_^−^ units, led to the formation of zeolite NaA. The metakaolin for the reaction was prepared from kaolin using a solvent-free calcination method. The sodium aluminate precursor was prepared either by calcination of a mixture of sodium hydroxide and γ-alumina or was made in situ by mechanochemical milling from sodium hydroxide reactant and added γ-alumina. Thus, the synthetic procedure provided by Prokof’ev has particular appeal in the context of solvent-free green chemistry. 

A similar methodology was applied for the synthesis of the zeolite LTA from metakaolin, sodium aluminate and excess γ-Al_2_O_3_ [169]. This synthesis of LTA zeolite is an interesting example of topochemical reaction control and structure templating in an inorganic reaction system. Prokof’ev and co-workers established that the milling/calcination process provides an optimum yield of LTA zeolite if the milling period is limited to a narrow time window between five and seven minutes. The explanation for this was found in a complex interplay of solid-state reactivity, polymorphism, and the structure-directing role of sodium aluminate during the calcination of the milling mixture. During short milling times, the sodium aluminate exists as a mixture of cubic and tetragonal forms, both of which exhibit lattice parameters compatible with the LTA zeolite structure. The heating of the reaction mixture containing these two sodium aluminate forms results in the formation of the porous zeolite LTA. Longer (e.g., 15 min) milling times lead to the complete conversion of sodium aluminate into an orthorhombic form that no longer displays a lattice size match to the LTA zeolite. The calcination of such a reaction mixture leads to the formation of the non-porous close-packed sodalite structure. The formation of zeolite LTA was found to occur also simply by the calcination of sodium aluminate and metakaolin at 600 °C, but in much lower yields than in the absence of mechanochemical treatment. These observations imply that the formation of the aluminosilicate framework by calcination is strongly topochemically controlled and that mechanochemical treatment has the important role of bringing the surfaces of reactant solids into close contact required for surface-controlled synthesis. The non-porous sodalite structure also resulted from calcination of milled reaction mixtures that contained water, either in the form of non-calcined kaolin or in the form of sodium tetrahydroxyaluminate formed in situ by replacing the excess γ-Al_2_O_3_ with Al(OH)_3_. The solvent-free synthesis of zeolites by a combination of mechanochemical and annealing treatment has also been reported by Ren and co-workers [170], and an extensive overview of mechanochemistry of zeolites has been provided by Rainer and Morris [171].

### 8.2. Ferrite Synthesis through Iron Oxide Generated via Aerobic Oxidation of the Milling Assembly

**Figure 10:**
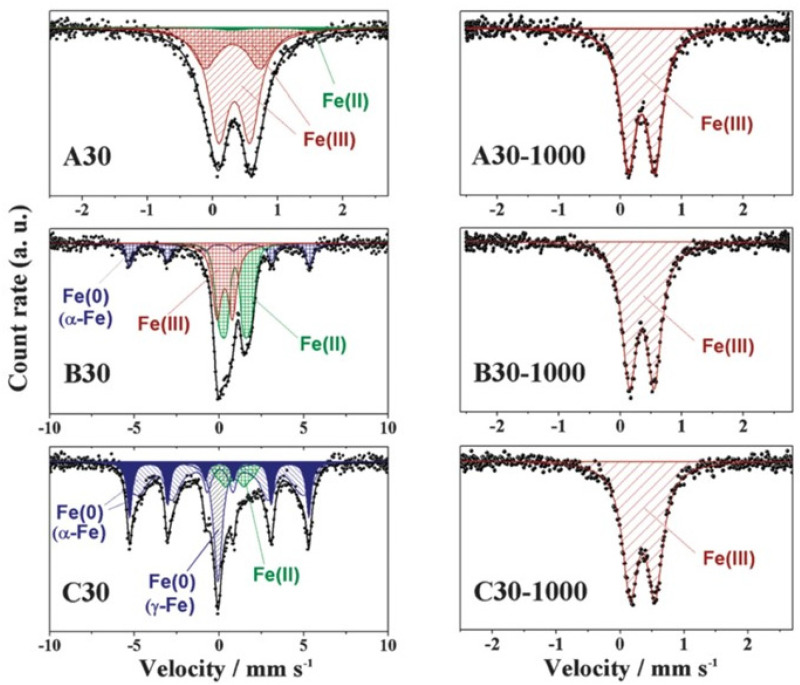
Mössbauer spectra of the samples of ZnO milled for 30 h in a chromium-rich stainless-steel bowl with stainless stell balls before (**left**) and after calcination at 1000 °C (**right**). Conditions: (A) exposed to air, with atmosphere refreshed every hour, (B) sealed under air, and (C) sealed under nitrogen [172].

An interesting observation combining reaction vessel contamination and reaction with oxygen from the air was reported by Štefanić et al., who studied the mechanochemically synthesis of ZnFeO_4_ from varied precursors under controlled atmosphere. Depending on the nature of the gas introduced: (A) under air, jar opened every hour to refresh the atmosphere; (B) under air at the beginning and never opened before the chosen reaction time; and (C) under nitrogen, the range of products obtained from milling pure zincite (ZnO) varied drastically [172]. The nature of the species forming was investigated by PXRD and Mössbauer spectroscopy, before and after calcination (Figure 10). When milling under repeated exposure to air, a progressive change of colour was observed from white (t = 0) to grey (t = 5 h) to orange (t = 14 h) and finally to black (30 h). After calcination at 500 or 1000 °C, no significant colour change was observed, matching the lack of structural change during calcination. The initial phase appearing after 1 h, in addition to zincite, is ferrite (α-Fe), likely due to iron leached from the reaction vessel, coherent with the grey colour observed. After 5 h of milling (with exposure to air every hour), the green colour observed matches that of the wüstite (FeO) observed by PXRD. From 9 h, the ferrite (ZnFe_2_O_4_) appears, likely through reaction between hematite (Fe_2_O_3_) and zincite (ZnO) and becomes the major phase past 14 h, with more resolved diffraction peaks after calcination at 1000 °C. Interestingly, no ferrite is observed under the other conditions (sealed under air or nitrogen) until the samples are calcinated. In conditions B, with exposure to air initially, the main impurity observed is wüstite (FeO) after 14 h, leading to a green product, turning black after further milling to 30 h total. Milling under inert conditions (C) lead to brown/black product at every time point, with PXRD pointing to both ferrite (α-Fe) and austenite (γ-Fe), presumably due to the different steel used for balls and bowl. Mössbauer spectroscopy of samples milled for 30 h then calcined (Figure 10) showed the range of oxidation states for iron described above, with mostly Fe(0) when using nitrogen, and increases in both Fe(II), forming initially to produce wüstite (FeO), and Fe(III), upon increasing the amount of air introduced during milling. The analysis of the iron origin based on different composition for bowl and balls reveals that the metallic iron particles released in conditions (C) lead to the increased abrasion of the bowl surface.

### 8.3. Mechanochemical Reactions with Gaseous Reagents: Synthesis of the Battery Material LiMn_2_O_4_

Whereas mechanochemically activated reactions in the presence of organic vapours, such as accelerated ageing and vapour-assisted tumbling have attracted considerable attention in the context of softer metal–organic (e.g., MOFs) and organic (e.g., cocrystals, organic reactions) materials [173], organic vapours can also be used to induce transformations of high-melting organic oxides. An example is the route for mechanochemical reduction of metal oxides described by Abe and Sano [174], who conducted a systematic exploration of the structural and chemical transformations of MnO_2_ upon milling in the presence of organic vapours. In particular, milling of MnO_2_ in an inert gas atmosphere which also contained acetone, ethylmethyl ketone, diethylketone, methanol, or ethanol was found to lead to the appearance of manganese(II) oxide, Mn_2_O_3_. The systematic step-by-step analysis [175] of the reaction involving acetone vapour using diverse methods, notably PXRD, X-ray photoelectron spectroscopy (XPS) and mass spectrometry, showed that Mn_2_O_3_ formation is preceded by the structural rearrangement of β- into γ-MnO_2_ (pyrolusite), followed by the oxidation of acetone into CO_2_ and acetaldehyde. The mechanochemical gas–solid reduction of MnO_2_ was further coupled in a one-pot process for the mechanosynthesis of the mixed-metal oxide LiMn(III)Mn(IV)O_2_ of relevance in battery technology: ball milling of MnO_2_ and either Li_2_CO_3_ or LiOH in the presence of acetone led to the formation of LiMn_2_O_4_ in a mixture with Mn_2_O_3_ and MnO_2_. Further heating to 700 °C led to complete transformation of the reaction mixture into LiMn_2_O_4_.

### 8.4. Boranes and Borohydrides

**Figure 11:**
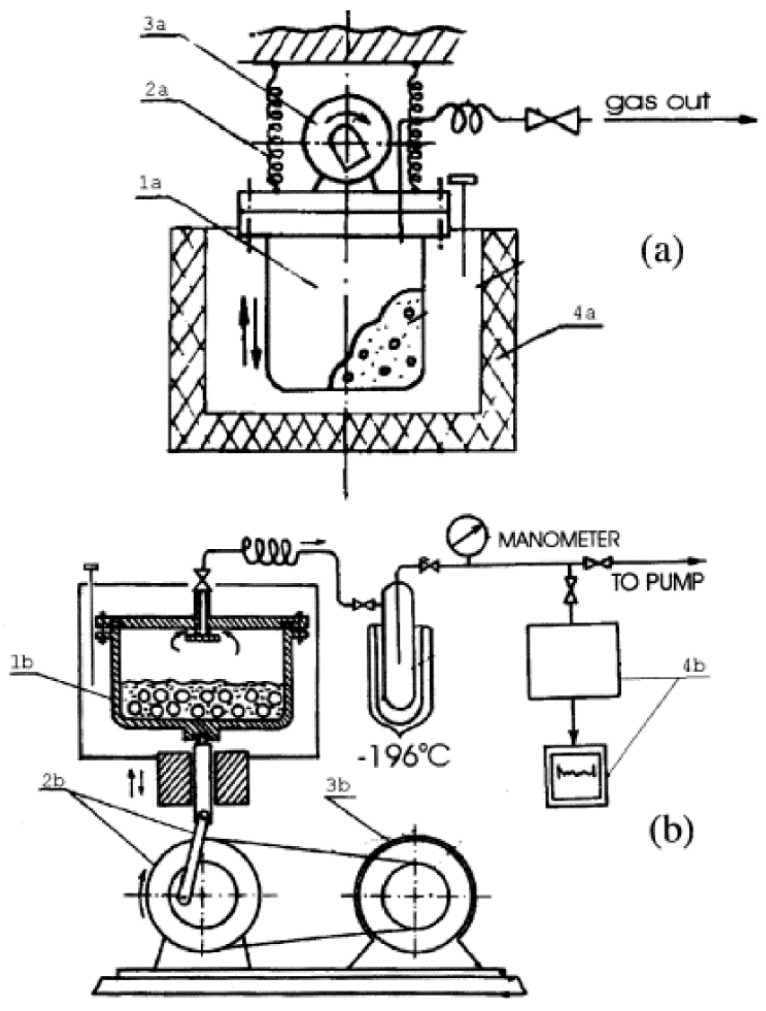
The schematic design of a vibratory hermetic ball mill for mechanochemical reactions, described by Schlesinger: (**a**) a mill with the inertial eccentric vibrator: 1a—mill body with the balls and reactants; 2a—elastic suspension support (springs); 3a—vibrator with the electric motor; 4a—Dewar vessel; (**b**) mill with the crank drive: 1b—mill body with the balls and reactants; 2b—crank drive of the vibrator; 3b—electric motor; 4b—automatic gas burette with recorder [177].

Whereas binary hydrides could, in principle, be synthesized by a direct reaction of hydrogen gas with a suitable element, such synthetic strategies are sometimes avoided due to technical and safety issues associated with handling gaseous hydrogen in a rapidly milled environment. Such difficulties are further augmented by the often highly reactive nature of the resulting hydride. This is best illustrated in the mechanochemical synthesis producing or involving highly flammable boranes and complex borohydrides, which often involve complex exchange and redox processes and for which a specialized hermetically sealed apparatus was developed by Volkov’s group [176]. According to Volkov, the first synthesis of a boron hydride through mechanochemical means was reported by Schlesinger, who prepared NaBH_4_ in up to 60% yield by reacting sodium hydride with boron oxide at a temperature over 300 °C in a rapidly rotating Pyrex tube containing fifteen ¼″ stainless steel ball bearings (Figure 11) [177]. The reaction proceeded also in the absence of ball milling but yielded only ca. 17% of NaBH_4_ expected based on sodium hydride. The simplest boron hydride, diborane gas, is readily obtained through a number of mechanochemical reactions, all of which involve the reaction of an alkaline metal borohydride with either a main group (Sn^II^, Pb^II^, Ge^IV^) or a transition metal (Cr^III^, Zn, Cd) halide or with iodine, yielding borane, the metal, and an alkaline metal halide. Of these reactions, particularly useful was the one involving SnCl_2_ (Equation (15)):

SnCl_2_ + 2 MBH_4_ → B_2_H_6_ + H_2_ + Sn + 2 MCl (15)

The non-hygroscopic nature of tin(II) chloride and the ease of converting Sn back to the SnCl_2_ reagent enabled this high-yielding reaction to be used for generating high yields (96–98%) of pure diborane, not contaminated with higher boranes or tin hydrides. The reaction could be readily optimized by varying the milling frequency (*f*) and amplitude (*A*) in this apparatus. The analogous reaction of germanium(IV) iodide is an interesting example of a mechanochemical reaction that follows a different route under mechanochemical conditions than using solution chemistry (Equations (16) and (17)):

GeI_4_ + 4 MBH_4_ → 2 B_2_H_6_ + Ge + 4 MI + 2 H_2_ (by milling) (16)

GeX_4_ + BH_4_^−^ + H_3_O^+^ → GeH_4_ + 4 X^−^ + H_2_ + B(OH)_3_ (in aqueous solution) (17)

The mechanochemical reaction of organoammonium salts with alkaline metal borohydrides allows the direct synthesis of borane adducts of primary, secondary, and ternary amines as well as pyridine. The syntheses of trimethylamine and triethylamine adducts of borane were also conducted at a scale of 100 g and more using a rotational ball mill. The chemical reaction involves an acid–base (or a redox) reaction and adduct formation (Equation (18)):

R_1_R_2_R_3_NH^+^Cl^−^ + MBH_4_ → MCl + R_1_R_2_R_3_N-BH_3_ + H_2_ (18)

The reactivity of the metal borohydrides increases in the sequence K < Na < Li, with the reactions involving LiBH_4_ achieving 96% yields within 5 min, those involving NaBH_4_ around 80% after 2 h, and those involving KBH_4_ yielding ca. 34% of the triethylamine adduct after 4 h of mechanochemical activation. The mechanochemical reaction was also readily applicable for this synthesis of the bis(borane) adduct of hydrazine by using hydrazine dihydrochloride or hydrazine sulphate as the starting material.

In 2006, Jeon and Cho demonstrated how mechanochemical milling provides a simple route to the synthesis of zinc borohydride, Zn(BH_4_)_2_ [178]. The simple milling of NaBH_4_ and ZnCl_2_ in a reaction jar charged in an argon atmosphere gave rise to the quantitative formation of Zn(BH_4_)_2_ within 30 min, as established by powder X-ray diffraction. However, the thermal analysis of the material indicated room-temperature instability with the formation of diborane. Whereas this particular study indicated that Zn(BH_4_)_2_ was unsuitable as hydrogen storage material, it is unclear whether its low stability is associated with mechanical treatment or the presence of the presumably inert NaCl by-product. Indeed, an effect of milling on the decomposition of a transition metal borohydride was observed by Gennari and co-workers, who systematically investigated the direct synthesis of zirconium borohydride Zr(BH_4_)_4_ by the milling of ZrCl_4_ with excess NaBH_4_ [179]. Although LiBH_4_ was observed to be more reactive than NaBH_4_ in this process, in agreement with the early observations made by Volkov, NaBH_4_ was selected as a more viable precursor. This choice was based on its significantly lower cost (roughly 1/10th of the cost of LiBH_4_ as well as on the fact that its hydrogen content (10.8% by weight) was similar to that of the expected product Zr(BH_4_)_4_ (10.7% by weight). This avoids the problem of converting an expensive and high hydrogen-content precursor (the weight fraction of hydrogen in LiBH_4_ is 18.4%) into a low-content product. The planetary milling of NaBH_4_ and ZrCl_4_ at a high speed of 400 rpm led to the nonquantitative yield of Zr(BH_4_)_4_ because of extensive diborane formation. Diborane formation could be reduced by conducting the reaction under milder conditions of 300 rpm and 200 rpm, but this also reduced the speed of the reaction. Milling for 6 h at 300 rpm was identified as optimum reaction conditions with respect to time and diborane formation, providing an approximately 55% yield of the highly volatile Zr(BH_4_)_4_. For comparison, the use of LiBH_4_ provided up to 91% reaction yield under the same conditions. The same procedure could readily be applied for the synthesis of a deuterated analogue, Zr(BD_4_)_4_, with the same limitations with respect to the rate of product formation and subsequent decomposition into borane.

### 8.5. Mixed Metal Hydrides and Borohydrides

Beginning in the late 1990s, the study of mechanochemical transformations of metal hydrides became increasingly interesting due to the perceived importance of such materials as hydrogen storage and release materials. Particular attention has been given to the combinations of hydrogen with other light elements, such as lithium, beryllium, magnesium, and aluminium. As demonstrated by selected examples given in this chapter, milling provides a convenient, solvent-free mechanochemical route to the synthesis of binary (e.g., MgH_2_) and complex (e.g., Mg(AlH_4_)_2_) metal hydrides, as well as a means to make their use for hydrogen storage more feasible by mechanical activation leading to lower temperatures of hydrogen gas release. A novel application of mechanochemical milling in the context of metal hydride chemistry is as a synthetic element in the high-throughput screening for novel hydrogen storage materials. In that way, the established use of mechanochemical milling in screening for pharmaceutical molecular materials has recently received an unexpected analogy in the chemistry of highly air-sensitive, metal-based inorganic materials.

### 8.6. Mechanochemical Activation and Transformation of LiAlH_4_

Lithium aluminium hydride was a target of a number of mechanochemical studies, presumably due to its commercial availability as well as popular use as a strong reduction agent in organic synthesis. Due to the inconsistencies of previous studies on the mechanochemical activation of LiAlH_4_, Balema and co-workers have explored the thermal stability of samples exposed to short (10 min) and increasingly long milling times (35, 75, and 100 h) in a helium atmosphere [180]. X-ray powder diffraction analysis demonstrated that, while brief milling did not noticeably influence the properties of the complex hydride, prolonged milling induced X-ray reflection broadening consistent with the anticipated particle size reduction and amorphization, as well as the decomposition to form metallic aluminium and Li_3_AlH_6_, presumably with the loss of gaseous H_2_ (Equation (19)).

3 LiAlH_4_ → Li_3_AlH_6_(s) + 2 Al(s) + 3 H_2_(g) (19)

A combination of thermal calorimetric and X-ray powder diffraction measurements indicated that the reaction goes almost to completion following 110 h of milling. The analysis of the resulting solid product revealed the presence of 1% by weight of iron, which presumably originated from the milling assembly. Indeed, the subsequent milling of commercial LiAlH_4_ in the presence of 10% by the weight of iron powder demonstrated the acceleration of decomposition reaction, such that 35 h milling yielded a product that resembled that obtained by the 75 h milling of pure LiAlH_4_. It is, therefore, clear that the previously observed inconsistencies in the mechanochemical behaviour of LiAlH_4_ are most likely related to the interaction with trace impurities coming from the milling assembly. 

Mechanochemical treatment was found to have a strong effect on the thermal decomposition of LiAlH_4_, which takes place first via the transformation into Li_3_AlH_6_, Al metal and hydrogen gas described by Equation (19), and then into lithium hydride (LiH), Al metal and more hydrogen gas (Equation (20)).

2 Li_3_AlH_6_ → 6 LiH(s) + 2 Al(s) + 3 H_2_(g) (20)

Mechanochemical milling reduced the temperature of thermal decompositions described by Equations (19) and (20), and the extent of this activation was related to the reduction of particle size in a systematic investigation by Andreasen and co-workers [181]. This work yielded activation energies for these two reactions of 80 kJ mol^−1^ and 100 kJ mol^−1^, respectively. The activation energy of only the first reaction was found to be significantly diminished by milling particle size reduction, clearly indicating this to be an important component of mechanochemical hydride activation. Such a conclusion is in agreement with the systematic studies that have been performed on nanoparticle-based samples of other metal hydrides, most notably NaAlH_4_ [182]. A subsequent study by Ares and co-workers also observed the decomposition of LiAlH_4_ upon milling, evidenced by the reduction in hydrogen capacity by 0.5% within the first 1.5 h of milling, and reaching approximately 1% within 7.5 h of milling [183]. At the same time, powder X-ray diffraction did not reveal any other crystalline phases except LiAlH_4_ in the milled material. This work identified two different effects of milling on the thermal stability of LiAlH_4_. At low milling times (under 14 h), both reactions described by Equations (19) and (20) were shifted to lower temperatures. The maximum shift was 60 °C, attained within 90 min of milling. At milling times longer than 14 h, the decomposition took place only via the reaction described in Equation (20), presumably because of Li_3_AlH_6_ now becoming the dominant sample component.

### 8.7. Catalytic Room-Temperature Dehydrogenation of LiAlH_4_

Catalytic room-temperature dehydrogenation of LiAlH_4_ upon milling to form Li_3_AlH_6_ was observed in the presence of a few mol percent of TiCl_4_ [184]. The high efficiency of this catalytic process is observed in the fact that the dehydrogenation transformation was complete within 5 min milling with only 3 mol% TiCl_4_. Milling is necessary for this catalytic hydrogenation, as demonstrated by manually mixing a catalytic amount of TiCl_4_ and LiAlH_4_ in a mortar under helium atmosphere: although the mixture turned grey (presumably due to the reduction of TiCl_4_ by the metal hydride reagent), powder X-ray diffraction did not indicate any trace of Li_3_AlH_6_ formation. A subsequent detailed study, conducted with temperature control involving forced air-cooling, revealed that the catalysis, initially presumed to be caused by the formation of TiH_2_, is actually related by the formation of trace amounts of a microcrystalline alloy Al_3_Ti [185]. Whereas the alloy is known to exist in two polymorphic modifications, the stable tetragonal and the metastable cubic one, no particular dependence of the catalytic effect on the polymorphic composition was observed. Consequently, the mechanochemical decomposition of LiAlH_4_ in the presence of TiCl_4_ represents an example of heterogenous catalysis during mechanical milling. Milling with the addition of catalytic amounts of simple binary halides of vanadium(III) (VCl_3_, VBr_3_) and aluminium (AlCl_3_) was found to reduce the temperature of both reactions of the thermal decomposition of LiAlH_4_ by ca. 25 °C [186]. The vanadium-based additives also enhanced the loss of hydrogen during mechanochemical treatment, while this effect was not observed in case of the AlCl_3_ additives. The mechanisms of mechanical activation using these metal halides are not yet known. Since the Rietveld analysis of diffraction data indicated that the particle sizes for LiAlH_4_ samples milled with (66–110 nm) and without (91 nm) the halide additives are similar, it was proposed that observed activation might be better explained by the possibly porous topology of resulting particles, rather than a reduction in their size.

Milling with transition (Ti, Sc) and rare-earth metal (Ce, Pr) halides has been demonstrated as a viable technique to synthesize the highly activated samples of NaAlH_4_ towards reversible hydrogen absorption and release [187]. Similarly, the activation of NaAlH_4_ with pre-prepared titanium Ti_13_ nanoclusters was performed by mechanical milling, leading to materials capable of reversible hydrogen release at rates comparable to those of commercial liquid fuels [188]. Although the role of the metal halide or the Ti-cluster that is being used as a dopant in the milling activation process remains unknown, synchrotron diffraction studies led to the proposal that the active component in reversible (de)hydrogenation is an intermediate amorphous phase Al_x_Ti_1−x_ involving zerovalent Al and Ti [189].

### 8.8. Mechanosynthesis of Complex Aluminium Hydrides

The metathesis-type reaction described by Volkov for the synthesis of metal tetrahydroborates is also applicable for the synthesis of metal tetrahydroaluminates (also known as alanates). The synthesis of alanates through conventional solution-based means is hindered by the difficulty of obtaining a completely solvent-free sample of the complex hydride required for thermal analysis. Consequently, the mechanochemical metathesis of LiAlH_4_ or NAlH_4_ with other metal halides provides an alternative route to complex metal hydrides, which circumvents the problems of trace solvents. An example of such a process is the synthesis of magnesium alanate (Equation (21)) [190]:

NaAlH_4_(s) + MgCl_2_(s) → Mg(AlH_4_)_2_ + 2 NaCl (21)

Mechanochemical metathesis allowed the synthesis of Mg(AlH_4_)_2_ at room temperature within 1 h, with a reaction mixture of one gram. The powder X-ray diffraction analysis of the mechanochemical product confirmed Mg(AlH_4_)_2_ as the only present crystalline phase and thermal analysis provided the opportunity to calculate the enthalpy associated with the first decomposition step of Mg(AlH_4_)_2_ (Equation (22)):

Mg(AlH_4_)_2_(s) → MgH_2_(s) + 2 Al(s) + 3 H_2_(g) (22)

Based on thermal data collected on the mechanochemical sample, the reaction enthalpy at 0 K was calculated to be −20 kJ mol^−1^, indicating that the reaction was not suitable for reversible hydrogen storage at feasible (i.e., close to ambient) temperatures. However, a subsequent study of the same reaction by Varin and co-workers [191], conducted on magnesium alanate samples obtained by mechanochemical metathesis by 5 h and 10 h milling, revealed variability in thermal behaviour that prevented the calculation of the reaction enthalpy or decision whether it should be positive or negative. The same study also revealed the partial decomposition of Mg(AlH_4_)_2_ into MgH_2_ simply upon prolonged mechanochemical treatment. In that way, the behaviour of Mg(AlH_4_)_2_ under mechanochemical conditions might display similarity to LiAlH_4_ [180,185].

### 8.9. Complex Aluminium Hydrides with Octahedral Coordination

A mechanochemical method to synthesize Li_3_AlH_6_, containing the octahedral AlH_6_^3−^ anion, from the much more common LiAlH_4_ based on the tetrahedral AlH_4_^−^ ion, was systematically explored by Balema and co-workers [184]. The synthesis of Li_3_AlH_6_ was achieved by the high-speed ball milling of LiAlH_4_ and LiH in a 1:2 respective molar ratio and in an inert atmosphere of helium. Within five hours, the solvent-free transformation into Li_3_AlH_6_ was fully accomplished, as established by powder X-ray diffraction and thermal analysis. In particular, the indexing of the X-ray diffraction pattern of the mechanochemical complex hydride yielded crystallographic cell parameters identical to those for Li_3_AlH_6_ previously obtained via an organic solvent-based method. The described synthesis of Li_3_AlH_6_ represents a mechanochemical pathway different from those observed for metal halides interacting with aluminium or boron complex hydrides (Equation (23)).

LiAlH_4_(s) + 2 LiH(s) → Li_3_AlH_6_(s) (23)

The thermal decomposition of mechanochemically prepared Li_3_AlH_6_ into Li, Al, and H_2_ takes place between 207 °C and 260 °C, in excellent agreement with the data obtained from the material using conventional means. A similar synthetic procedure was performed to prepare the deuterated sodium analogue of Li_3_AlH_6_. The milling of deuterium-based LiAlD_4_ with NaD or NaH has been used for the synthesis of LiNa_2_AlD_6_ and LiNa_2_AlD_4_H_2_, with the former subsequently having been structurally characterized using synchrotron X-ray powder diffraction [192].

### 8.10. Mechanochemistry of Magnesium Borohydride

Because of its low cost and low atomic weight, magnesium and magnesium alloys are among the most prominent materials for hydrogen storage. Some of the details of magnesium hydride (MgH_2_) mechanosynthesis from the elements have already been provided above along with highlight of the mechanochemistry of magnesium alanate (Mg(AlH_4_)_2_). Suffice it to say that in both cases, the ease of hydrogen binding and the reverse process of hydrogen gas release are improved for mechanochemically synthesized systems, most likely due to increased surface areas and small particle sizes. A similar effect is also observed for magnesium borohydride, Mg(BH_4_)_2_, the recently introduced third promising member or the magnesium hydride-based family of materials for reversible hydrogen storage. It has long been known that the thermal decomposition of Mg(BH_4_)_2_ leads to the formation of magnesium boride (MgB_2_) and the release of ca. 14% by weight of H_2_. The analysis of reaction enthalpies suggested that, although the dehydrogenation of Mg(BH_4_)_2_ is very favourable, it should not preclude the ability for the reverse reaction under moderate conditions. The first successful hydrogenation of magnesium borohydride was reported by Severa and co-workers who demonstrated up to 75% transformation of MgB_2_ into Mg(BH_4_)_2_ by the mechanochemical activation of MgB_2_ starting material and its subsequent isothermal reaction with hydrogen gas at 950 bar and 400 °C [193]. The 75% conversion proceeded without the significant formation of the complex borohydride anions deleterious for the reaction and allowed the release of >11% hydrogen by weight.

Since mechanical activation was central to enabling the hydrogenation of magnesium boride in the procedure of Severa et al., it is reasonable to assume that the isothermal high temperature treatment might actually be limiting the efficiency of hydrogenation by thermally annealing the activated MgB_2_. Indeed, a subsequent study by Gupta and co-workers demonstrated the complete hydrogenation of MgB_2_ into Mg(BH_4_)_2_ upon mechanical treatment at room temperature and hydrogen gas pressures between 50 bar and 350 bar [194]. Higher ball-to-sample weight ratios (up to 160) and higher hydrogen gas pressures while milling improved the degree of conversion into Mg(BH_4_)_2_. The improvement was most likely due to a greater efficiency in breaking down the anionic borohydride cluster anions and the hexagonal boride anion layers in MgB_2_. The thermal dehydrogenation of the mechanochemically synthesized Mg(BH_4_)_2_ was conducted at temperatures up to 390 °C, which allowed the release of up to 4% by weight of H_2_ gas. Such thermal treatment led to the almost exclusive production of MgB_2_, which could again be mechanochemically hydrogenated at room temperature in order to provide a material with ca. 90% original hydrogen storage capacity.

### 8.11. Screening for New Hydride and Hydrogen Release Materials by Mechanochemistry

A very recent development in the application of milling mechanochemistry for developing hydrogen storage materials is mechanochemical screening. A high-throughput methodology to screen for hydrogen storage materials was demonstrated by Li and co-workers, who reported “incorporating parallel gas sorption instruments and parallel ball mills into a complete high throughput workflow capable of synthesizing and analysing >200 powder samples per day” [195]. Although a more detailed description of the instrumentation was not provided, its use was illustrated on the model system of lithium magnesium imide, a material which is known to absorb up to 5.6% of its weight of hydrogen gas through the reversible reaction (Equation (24)):

Li_2_Mg(NH)_2_(s) + 2 H_2_(g) ↔ 2 LiH(s) + Mg(NH_2_)_2_ (24)

Through screening binary and ternary mixtures of Li_2_Mg(NH)_2_ with over 50 additives capable of acting as starting material dopants, catalysts, inhibitors, or process control agents for the hydrogen uptake reaction, Li and co-workers identified the mixture containing 5% LiBH_4_ and 0.5% La as the optimum one with respect to the rate of hydrogen absorption, hydrogen capacity, and cycle life. It is difficult to imagine how this particular mixture, which provides a steady hydrogen sorption and release capacity of ca. 4.5% throughout at least 11 hydrogenation/dehydrogenation cycles, could have been discovered through a direct search, illustrating the value of high-throughput mechanochemistry-based screening for the discovery of new materials.

A different take on the mechanochemical screening of hydride materials was demonstrated by Ravnsbæk and co-workers, who conducted a screen of metal borohydrides by milling together cadmium chloride (CdCl_2_) with either LiBH_4_, NaBH_4_, or KBH_4_ in different stoichiometric compositions [196]. The mechanochemical experiments were followed by thermal treatment during which variable temperature X-ray powder diffraction data were collected. The altogether 875 collected powder X-ray diffraction patterns revealed the presence of ten known phases, as well as four new crystal structures. The new crystal structures of α- and β-forms of Cd(BH_4_)_2_, K_2_Cd(BH_4_)_2_, and KCd(BH_4_)_3_ were directly determined from X-ray powder diffraction data. It was noted that the yield of cadmium borohydrides was lowered by the side reaction forming complex chlorocadmates from alkaline metal chlorides and CdCl_2_, for example, following the Equation (25):

3 KBH_4_ + 3 CdCl_2_ → KCd(BH_4_)_3_ + 2 KCdCl_3_ (25)

While our understanding of these reactions has improved over the past decades, further investigations are still required. A promising methodology for the studies of the study of the preparation and reactivity of hydride species with potential for hydrogen release is to follow these mechanochemical reaction in situ using spectroscopic methods, such as Raman spectroscopy [197,198]. Biliškov and coworkers recently demonstrated the potential of this method when studying the mechanochemical preparation of complex amidoborane salts: Li_2_Mg(NH_2_BH_3_)_4_ and Na_2_Mg(NH_2_BH_3_)_4_ [199], a class of compounds heavily studied as a potential material of interest for hydrogen due to their low molecular weight leading to high gravimetric and volumetric hydrogen content [200].

## 9. Characterization Methods

**Figure 12:**
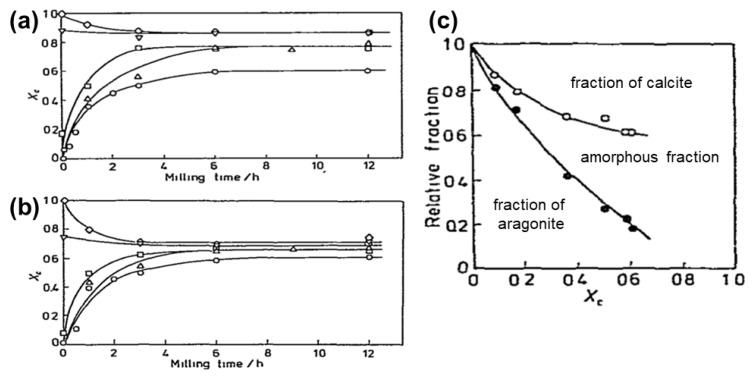
Mechanochemical equilibration of calcite and aragonite, studied by Iguchi and Senna: (**a**) change in calcite content for different milled samples based on PXRD analysis and (**b**) based on infrared spectroscopy analysis. Different symbols are used to distinguish data collected for samples annealed at different temperatures (between 473 and 773 K). (**c**) Change in the relative contents of calcite, aragonite, and amorphous CaCO_3_ phases with respect to fraction of calcite [202]. Fractions of calcite and aragonite are designated by open and full circles, respectively.

A broad overview of methods used in the product characterization and reaction monitoring in mechanochemistry was recently provided by Julien et al. [175], while more focused reviews on real-time, in situ reaction monitoring techniques have been provided by our team [197], as well as by Petersen and Weidenthaler [201] and by Michalchuk and Emmerling [198]. Consequently, provided herein is only a brief highlight of several representative techniques used in characterizing inorganic mechanochemical reactions and products. In general, the reaction monitoring and characterization of products and intermediates in inorganic mechanosynthesis encompasses a diversity of techniques, from X-ray powder and single crystal diffraction, scanning and transmission electron microscopies (SEM, TEM, respectively), to a very wide range of spectroscopic techniques, such as infrared, Raman and visible fluorescence emission spectroscopy, solid-state nuclear magnetic resonance spectroscopy (ssNMR), Mössbauer spectroscopy, as well as other types of analyses, notably thermal methods and surface area measurements. Generally, combining the different types of analytical techniques is important to achieving a clear understanding of mechanochemical products and reactivity. For example, Iguchi and Senna have used infrared spectroscopy to validate the results of PXRD analysis in their investigation of the mechanochemical equilibration of polymorphs of CaCO_3_ (Figure 12) [202]. This archetypal example of mechanochemical equilibration, initially studied by Schrader and Hoffmann, was found to be mediated by an amorphous phase [203].

**Figure 13:**
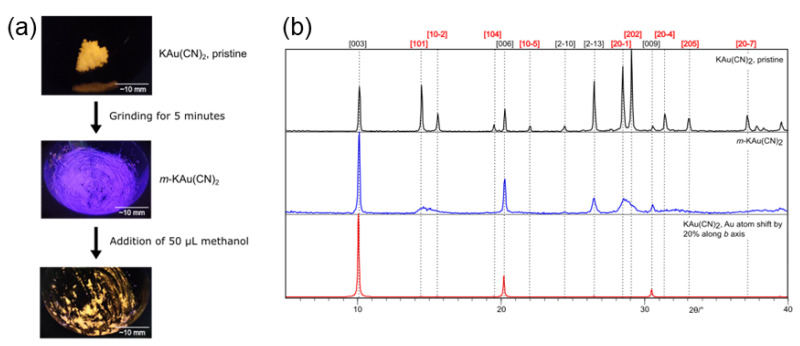
Mechanochemical interconversion of polymorphs of KAu(CN)_2_: (**a**) appearance of a sample of KAu(CN)_2_ before and after grinding, and after the addition of a small amount of methanol under ultraviolet light; (**b**) comparison of PXRD patterns (top-to-bottom) of a pristine sample of KAu(CN)_2_, a sample of *m*-KAu(CN)_2_ obtained by grinding and simulated for the structure of KAu(CN)_2_ modified to represent disordering of Au(CN)_2_^−^ ions [204].

As another example of using complementary diffraction and spectroscopic techniques, the combination of PXRD analysis and fluorescence emission was used by Vainauskas and co-workers for the observation of a new polymorph of the historically and technologically important salt KAu(CN)_2_ [204]. The milling or manual grinding of the commercially obtained material led to the broadening and/or disappearance of specific X-ray reflections, which was interpreted through a proposed turbostratic disordering of dicyanoaurate(I) ions. Notably, this form of the salt, termed *m*-KAu(CN)_2_ was found to also exhibit a significantly different fluorescence emission spectrum upon irradiation with ultraviolet light, switching from initially orange to violet, corresponding to change in the emission maximum from 376 nm to 415 nm. Importantly, the changes were found to be completely reversible upon the addition of a small amount of solvent, such as methanol (Figure 13).

A combination of PXRD and thermogravimetric analysis (TGA), as well as other methods, have been used by Gotor and co-workers in studying the mechanochemical synthesis of titanium nitride (TiN) by milling titanium metal in nitrogen gas [82]. The comparison of PXRD and TGA data indicated that the reaction proceeds via an initial activation process, involving the formation of a solid solution of nitrogen in titanium, which subsequently crystallizes to yield TiN.

In contrast to the mechanochemical transformations of organic and metal–organic solids, where the recently emergent techniques for real-time and in situ reaction monitoring based on synchrotron PXRD and spectroscopy have been rapidly taken up and led to significant advances in mechanistic understanding, the adoption of such techniques in inorganic mechanochemistry has been slow [205,206,207]. An example of the real-time synchrotron PXRD monitoring of an inorganic transformation is the work by the Weidenthaler group, who observed the formation and polymorphic transformations of ZnS in real time [67]. Recently, the de Oliveira and Emmerling groups have introduced a tandem technique for real-time synchrotron X-ray diffraction and X-ray absorption spectroscopy (XAS) monitoring of mechanochemical reactions [208]. The methodology was first used for the real-time observation of the reduction of hydrated tetrachloroauric(III) acid (HAuCl_4_·3H_2_O) with different reducing agents: hydroquinone, ascorbic acid, and NaBH_4_. This work was subsequently complemented through electron microscopy imaging, not through real-time monitoring but through the step-by-step observation of ageing of mechanically activated samples [209].

**Figure 14:**
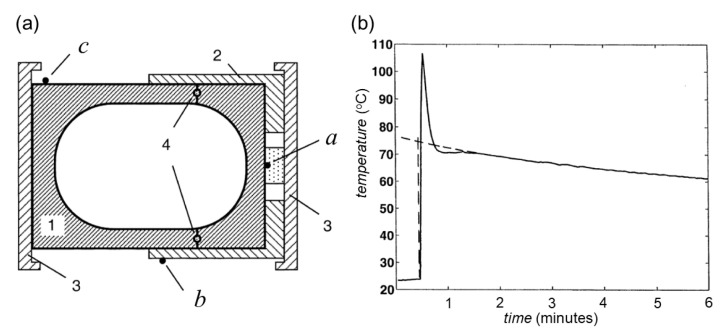
Real-time thermal monitoring of the course of the MSR of the reduction of Fe_3_O_4_ by milling with Al metal: (**a**) the milling vessel equipped with thermocouples situated at positions *a*, *b*, and *c*; (**b**) time-dependent temperature profile measured at position *a*. Components of the milling vessel: 1—steel body; 2—aluminium screw-on cap; 3—holding clamps; 4—O-ring seal [68].

Overall, however, the real-time monitoring of inorganic mechanochemistry has largely relied on recording the changes in pressure and/or temperature of the reaction mixture, which can provide valuable information on highly exothermic MSRs, as well as reactions that involve the sorption or loss of gases. An example is the use of a specially modified milling vessel with differently distributed thermocouples, described by Takacs, which enabled the thermal monitoring of the MSR of the reduction of Fe_3_O_4_ by milling with metallic aluminium (Figure 14) [68]. While traditionally associated with monitoring the mechanochemical transformations of inorganic substances, both pressure and temperature monitoring have recently been applied in the following transformations of metal–organic substances. For example, an advanced, more sensitive design for following the evolution of temperature in a mechanochemical reaction was reported by Užarević and co-workers [210]. This design enabled the monitoring of reactions that are less exothermic, such as the formation and polymorph interconversions of zeolitic imidazolate frameworks (ZIFs) from zinc oxide. Similarly, pressure-monitoring has recently also been applied to monitor the course of the synthesis of ZIFs from basic zinc carbonate, as well as the reaction of nascent material with CO_2_ gas [53,211].

## 10. Conclusions

We have provided a systematic overview of the mechanochemical reactions of inorganic substances, an area that has so far been addressed by a relatively small number of reviews compared to the abundance of literature providing overviews of mechanochemistry in organic synthesis, organic, metal–organic or hybrid inorganic–organic solid-state materials. We hope that this review, in which we have attempted to provide a broad cross-section of different types or materials and chemistries, will provide both a useful reference, as well as an inspiration, to inorganic chemistry researchers and materials scientists interested in mechanochemistry. At the same time, the herein proposed systematic categorisation of reactivity should be useful in the further development of the mechanochemical approaches to inorganic systems. The proposed systematic categorisation is by no means perfect, and it is likely to be modified by the continuous introduction of new, advanced technologies for conducting mechanochemical processes [212], including the development of equipment that enables reactions with gases [34], reactions at controlled temperatures, photochemical reactions [213], transformations in the absence of milling media, such as SpeedMixing [214] or resonant acoustic mixing (RAM) [215], as well as by the development of a clearer mechanistic and theoretical understanding of mechanochemical processes [44]. With the expectation of many new innovations and advances in inorganic mechanochemistry, and in particular, new mechanistic knowledge offered through the implementation of real-time monitoring techniques based on powder X-ray diffraction and diverse spectroscopies [205,206,207,208], we hope that the current reviews and proposed categorisation will be a useful steppingstone for the further efficient and systematic growth of the field. 

## Data Availability

There is no data associated with this article.
